# Local and landscape scale determinants of macroinvertebrate assemblages and their conservation value in ponds across an urban land-use gradient

**DOI:** 10.1007/s10531-016-1286-4

**Published:** 2017-01-17

**Authors:** Ian Thornhill, Lesley Batty, Russell G. Death, Nikolai R. Friberg, Mark E. Ledger

**Affiliations:** 1 School of Geography, Earth and Environmental Sciences, University of Birmingham, Edgbaston, Birmingham, West Midlands B15 2TT UK; 2 Institute of Agriculture and Environment - Ecology, Private Bag 11-222, Palmerston North, 4442 New Zealand; 3 Norwegian Institute for Water Research (NIVA), Gaustadalléen 21, 0349 Oslo, Norway; 4 School of Geography, University of Leeds, Leeds, LS2 9JT UK

**Keywords:** Biodiversity, Urbanisation, Water quality, Machine learning, Aquatic ecology

## Abstract

Urbanisation represents a growing threat to natural communities across the globe. Small aquatic habitats such as ponds are especially vulnerable and are often poorly protected by legislation. Many ponds are threatened by development and pollution from the surrounding landscape, yet their biodiversity and conservation value remain poorly described. Here we report the results of a survey of 30 ponds along an urban land-use gradient in the West Midlands, UK. We outline the environmental conditions of these urban ponds to identify which local and landscape scale environmental variables determine the biodiversity and conservation value of the macroinvertebrate assemblages in the ponds. Cluster analysis identified four groups of ponds with contrasting macroinvertebrate assemblages reflecting differences in macrophyte cover, nutrient status, riparian shading, the nature of the pond edge, surrounding land-use and the availability of other wetland habitats. Pond conservation status varied markedly across the sites. The richest macroinvertebrate assemblages with high conservation value were found in ponds with complex macrophyte stands and floating vegetation with low nutrient concentrations and little surrounding urban land. The most impoverished assemblages were found in highly urban ponds with hard-engineered edges, heavy shading and nutrient rich waters. A random forest classification model revealed that local factors usually had primacy over landscape scale factors in determining pond conservation value, and constitute a priority focus for management.

**Electronic supplementary material:**

The online version of this article (doi:10.1007/s10531-016-1286-4) contains supplementary material, which is available to authorized users.

## Introduction

Worldwide, urban centres are expanding to accommodate an ever increasing human population (Grimm et al. 2008). As urbanisation intensifies, natural habitats are being destroyed by a range of anthropogenic pressures (McDonnell and Hahs 2008). Blue spaces such as rivers, streams and ponds are renowned for their ecologically diverse communities, and yet they are increasingly threatened by development and polluting runoff from impermeable catchments (Paul and Meyer 2001; Walsh et al. 2005). Previous urban–rural gradient studies have shown how the loss and fragmentation of patches (Medley et al. 1995) can reduce the diversity of a variety of groups, including birds (Goldstein et al. 1986), insects (McIntyre 2000), reptiles, mammals and amphibians (Dickman 1987). Biota in pond ecosystems are among the most vulnerable to urbanisation because these habitats are spatially isolated, small (<2 ha), and poorly protected by environmental monitoring programmes underpinned by the EU Water Framework Directive (Indermuehle et al. 2008).

Urban development can result in the creation of ponds for Sustainable urban Drainage Schemes (SuDS, Briers, 2014), highway water retention (Le Viol et al. 2009), industrial activity (Wood and Barker 2000) and aesthetic and amenity purposes. However, the rate of pond creation is frequently far outweighed by the rate of pond destruction, although questions remain as to whether these losses may be partially offset by a proliferation of small garden ponds, the number of which is still largely unknown. In the UK, 32% of ponds are estimated to have been lost over 120 years between 1880 and 2000 with the greatest losses occurring in urban areas (Biggs et al. 2005). In London, over 90% of ponds were lost between 1870 and 1984 (Langton 1985) whereas in Birmingham over 80% were lost between 1904 and 2009 (Thornhill 2013). Research is urgently needed to determine the biodiversity and conservation value of these neglected and increasingly threatened habitats.

Several studies have shown that ponds can contribute more to regional biodiversity than running waters (Williams et al. 2004; Davies et al. 2008) and whilst only a few ecological surveys have been conducted in urban ponds, they reveal that their biodiversity and conservation value can match their rural counterparts (Scher and Thièry 2005; Gledhill et al. 2008; Vermonden et al. 2009; Hassall and Anderson 2014; Hill et al. 2015), potentially due to a unique community adapted to highly modified landscapes (Hill et al. 2016). Nevertheless, urban pollution may reduce species richness in ponds, thereby increasing opportunities for non-native and or invasive species (Duguay et al. 2006; McKinney 2008), especially when combined with other factors particular to urban settings (e.g. garden escapes). Habitat degradation may also lead to biological homogenization across the wider pond network (McKinney 2006). The loss of ponds from the landscape can increase the isolation of remaining ponds, and in so doing undermine the dispersal processes essential for species persistence across urban landscapes.

Ponds are naturally isolated systems that rely on a frequent exchange of members of their ecological communities (Jeffries 1994; Briers and Warren 2000). They are thus recognised as metacommunities in which the shape of any one local community is contingent upon the relative influence of various factors acting at local and landscape scales (Leibold et al. 2004). Several local-scale factors can potentially shape macroinvertebrate assemblages in ponds, including concentrations of macronutrients (Declerck et al. 2005), pH (Biggs et al. 2005), riparian shading (Gee et al. 1997; Hassall et al. 2011), pond surface area (Bronmark 1985; Heino 2000), habitat complexity (Bronmark 1985; Declerck et al. 2005) and fish (Fairchild et al. 2000; Scheffer et al. 2006). Local factors may combine and interact to shape pond communities; for instance the effect of increased nutrient concentrations on macroinvertebrate richness is likely mediated by the presence of macrophytes (Scheffer et al. 1993; Declerck et al. 2005). Similarly, landscape-scale factors such as surrounding land-use may influence resilience to diffuse pollution (e.g. agricultural run-off) whereas the density of neighbouring ponds as colonist sources may govern rates of dispersal between habitat patches (Bilton et al. 2001).

Recent studies have assessed how landscape scale factors might influence pond ecological communities alongside local factors (Pellet et al. 2004; Declerck et al. 2006; Schmidt et al. 2008; Akasaka et al. 2010). Several studies use a data analytic approach incorporating multiple spatial extents (i.e. concentric ring analysis) to determine the spatial extent of the influence of the surrounding landscape on focal habitats and communities. They reveal that freshwater systems are typically influenced by the land-use of the immediate surroundings i.e. the first few hundred metres from the pond edge (Declerck et al. 2006; Akasaka et al. 2010). Few studies, however, result in clear guidance on how to prioritise management action to best improve biodiversity in urban ponds. This is particularly relevant as there is a pressing need for conservation to rely less heavily upon statutorily protected areas and move to the sustainable use of alternative biodiversity resources (Chester and Robson 2013). Thus urban conservation action must be cost-effective where management decisions are subject to many competing interests (Ahern 2013; Shwartz et al. 2014).

As small isolated habitats, ponds can be a logistically feasible focus for nature conservation (De Meester et al. 2005; Boix et al. 2012), yet the conservation value of resident biota is often poorly described or unknown. The resilience of biota in threatened habitats may be enhanced by careful management of habitat and water quality but further research is needed to identify the extent to which stressors affect pond biota in urban areas. In this paper, we report the results of a survey of 30 ponds across a land-use gradient conducted to (1) evaluate the biodiversity and conservation value of ponds in the West Midlands, UK, and (2) determine the key factors affecting pond communities, in order to better target management options for conservation. We expected that ponds, as isolated habitats, would contain markedly contrasting communities reflecting the wide range of stressors acting across the complex urban landscape. A concentric ring analysis of land-use was conducted to establish the spatial extent at which surrounding land-use most strongly influenced the taxonomic composition of macroinvertebrate assemblages. We hypothesized that macroinvertebrate assemblages would be most strongly related to landscape-scale factors at a short distance from the pond edge. Local physicochemical and landscape scale land-use data were then modelled to establish the relative importance of local and landscape-scale factors as determinants of macroinvertebrate assemblage composition in ponds, and to determine whether pond types could be identified across the dataset.

## Methods

### Study area

**Fig. 1:**
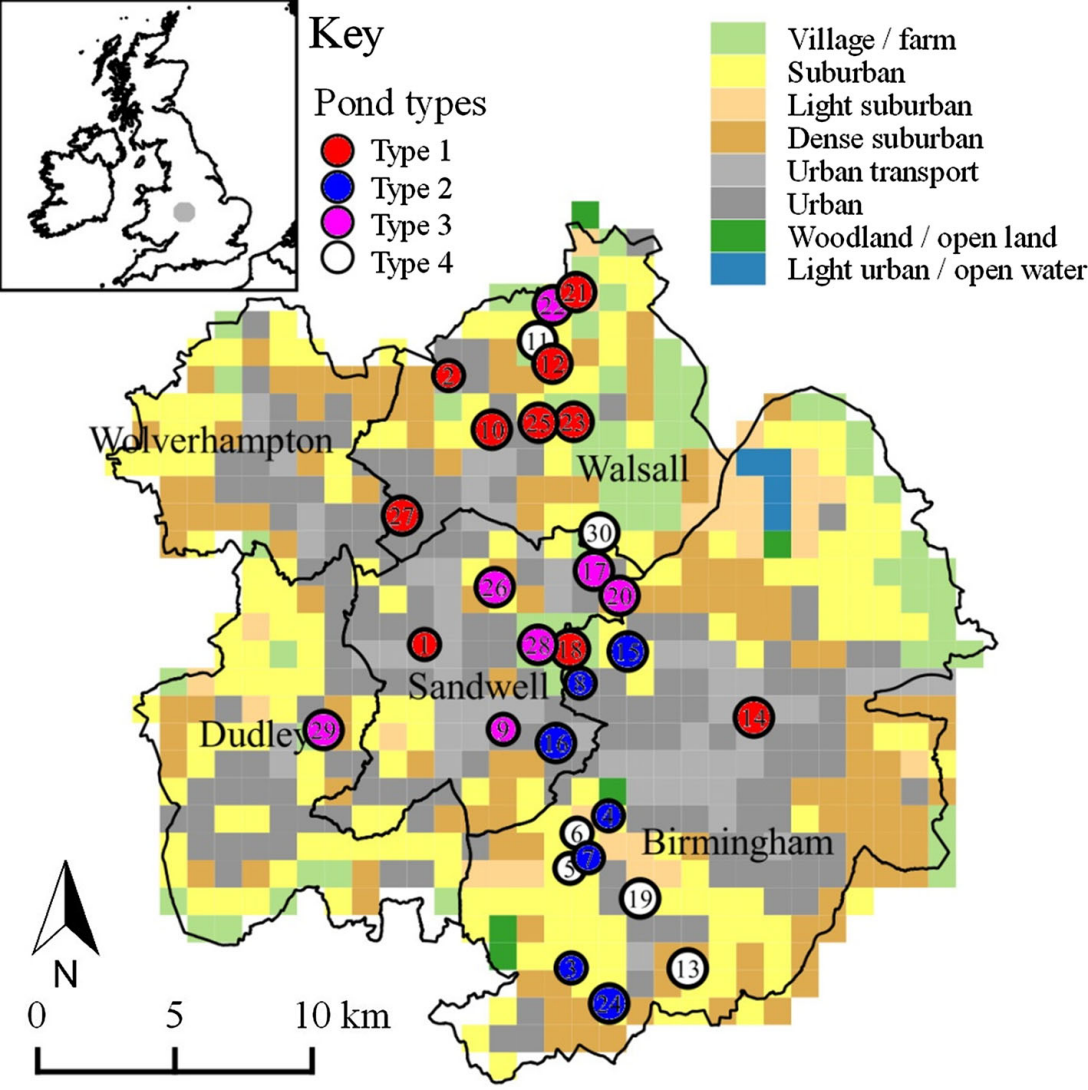
Geographic location of 30 study ponds (*circles*) in the West Midland conurbation, UK Sites are shown in relation to land-use within 1 km squares, using the classification of Owen et al. (2006). Pond types were established using a Ward’s hierarchical clustering procedure

The West Midlands conurbation of Birmingham, Sandwell, Dudley, Walsall and Wolverhampton (625 km^2^; 2.2 M inhabitants) is a central region of the UK (Fig. 1). The area has a rich industrial history of mining and manufacturing. Land-use is now predominantly suburban (55% cover), urban or industrial (19%), green space (16%) including parks and gardens and blue spaces (0.6%) such as flowing and still freshwaters (data derived from Land Cover Map 2007). Ponds are widely dispersed across the region and encompass small garden ponds, storm water basins, shallow naturalised wetlands, ex-marl pits and concrete-lined ornamental ponds within parks. For this study, we selected 30 ponds that hold water for at least four consecutive months of the year, between 2 m^2^ and 20,000 m^2^ (2 ha) in surface area (Pond Conservation Group 1993) from 1023 possible sites identified using a ArcGIS 9.3 Geographical Information System (GIS). Site selection was stratified across a gradient of eight urban land-use classes from rural villages to heavily urbanised city centres, characteristic of the West Midlands (Owen et al. 2006, Fig. 1). On average, study ponds were 10 km distant (min. 203 m, max. 25,935 m). Each pond was sampled in May–June 2009 and again in August 2009 with at least a two month period between samples (Biggs et al. 1998).

#### Macroinvertebrate sampling

Sampling was based on the standard protocols of the UK National Pond Survey (Biggs et al. 1998). A sample consisted of three-minutes of plunging/sweeping with a standard Freshwater Biological Association pond net (25 cm diameter, 1 mm mesh), with sampling effort divided equally among pond mesohabitat patch types (e.g. emergent macrophytes, submerged macrophytes, fine sediment). Samples were preserved in 70% industrial methylated spirit and later sorted from debris, identified to the lowest practicable taxonomic unit (usually species) and counted. Macroinvertebrate data from the two sampling periods were pooled to create a single taxon list for each pond (Biggs et al. 1998).

#### Local environmental variables

The concentration of major ions and trace metals was determined from 50 ml water samples collected from just below the water’s surface at the inflow, outflow and midpoint of each pond on each sampling occasion. Samples were filtered (1.2 μm Whatman GF/C) and stored at −20 °C with samples for trace metals acidified to pH 2 using nitric acid. Anions measured using ion chromatography (Dionex ICS2000, Dionex Corporation, Sunnyvale, AC, USA) were chloride, nitrate, phosphate and sulphate. Cations measured with a Dionex DX500 (Dionex Corporation, Sunnyvale, AC, USA) were sodium, ammonia, potassium, magnesium and calcium. Alkalinity was determined by titration (to pH 4.5; HACH, Dusseldorf, Germany). Trace metals (Co, Cr, Cu, Fe, Mg, Mn, Ni, Pb, Zn) were quantified by atomic absorption spectrometry (Perkin Elmer AA300, Perkin Elmer, Massachusettes, USA). The mean concentration of three measures was calculated for each sampling event.

Suspended solids and chlorophyll *a* were determined from a 5L water sample collected from each site on each sampling occasion collected as per chemical analyses. Suspended solids were determined as the freeze-dried mass (mg) of material filtered (Whatman GF/C, 1.2 μm pore size) from each sample. Chlorophyll *a* (mg/L) was determined spectrophotometrically from the filtered material using standard methods as outlined in Jeffrey and Humphrey (1975). Electrical conductivity (µS/cm), dissolved oxygen (% saturation) and pH were also measured (in triplicate) in the field using a YSI 556 handheld multi-probe meter, calibrated daily before use (YSI, Yellow Springs, OH, USA).

A suite of physical parameters were recorded in the field or calculated in GIS. These were pond surface area (ha) and the percentage of area classified as being open water, shaded by trees, fringing (e.g. *Typha* spp.) or floating vegetation (e.g. *Nymphaea* spp.), and percentage of hard-engineered pond bank (e.g. concrete). Surface area was calculated after the first spring 2009 sampling period using aerial imagery and field notes, with measures of water depth in fixed locations taken in order to assess seasonal changes in water depth and extent. Stakeholder consultations and site investigations determined water sources (i.e. stream inflows, groundwater, impermeable surfaces or building run-off) and the presence-absence of fish. Water level fluctuation (WLFI) was quantified as the standard deviation of water depth at fixed points sampled during spring, summer and autumn. Total macrophyte richness (presence-absence) was determined as the pooled occurrence of plant types within mesohabitats identified for each pond following the NPS methodology (Biggs et al. 1998). Macrophytes (emergent, submerged, floating and free floating) were identified to species level (after Haslam et al. 1995) with fine leaved specimens identified under microscope.

#### Landscape variables

A GIS combining five layers of spatial information, Ordnance Survey (OS) MasterMap, Land Cover Map 2007 (LCM2007), photogrammetry, Normalised Difference Vegetation Index (NDVI) was used to generate detailed land-use data. Landscape-scale variables were included to characterise surrounding land-use and connectivity to neighbouring wetland habitats. Metrics for land-use variables were calculated at 50, 100, 250, 500, 1,000 and 2,500 m from the pond edge to be incorporated into a concentric ring analysis with connectivity metrics calculated at 500 m (Waterkeyn et al. 2008) (Table 1).

**Table 1:** Land-use variables used within a concentric ring analysis and connectivity metrics, their sources and ranges generated from a combination of spatial layers within a GIS

Variable	Source (s)	Mean (max.–min.)
Land-use (50–2500 m)		
Impermeable surfaces	OS MasterMap	17.7 (0.00–31.0)
Improved grassland	Land Cover Map (2007)	17.8 (0.00–59.3)
Scrub (<3 m)	NDVI + photogrammetry	20.3 (10.2–44.8)
Trees (>3 m)	NDVI + photogrammetry	20.6 (4.8–52.2)
Connectivity (at 500 m)		
Number of ponds	OS MasterMap + aerial imagery	1.93 (0.0–4.0)
Coverage of aquatic habitat	OS MasterMap	13.2 (2.5–31.5)
Coverage of pond habitat	OS MasterMap + aerial imagery	5.5 (0.0–17.9)

#### Statistical methods

Ordination and cluster analyses are frequently used in ecological studies as they highlight patterns of association worthy of further investigation. Abundance data were log (*n* + 1) transformed before using Ward’s hierarchical, agglomerative clustering process (Murtagh and Legendre 2014) and Bray–Curtis similarity measure to identify macroinvertebrate assemblages. We then tested for statistically significant differences among observed clusters using one-way analysis of similarity (ANOSIM, 9999 permutations) and ordinated using non-metric multidimensional scaling (nMDS) in the R package (R Core Team 2016) ‘vegan’ (Oksanen et al. 2015). The ANOSIM test statistic ranges between 0 and 1 with a score closer to 1 indicating that all dissimilarities between clusters are larger than any dissimilarity among samples within each cluster (Clarke and Warwick 2001).

IndVal analysis (Dufrene and Legendre 1997) was performed to identify indicator species within each assemblage derived from the cluster analysis using the R package ‘labdsv’ (Roberts 2015). This analysis estimates the indicator value based on the relative abundance and occurrence frequency of each species in each previously defined cluster.

The conservation value of individual ponds was assessed using the Community Conservation Index (CCI), which accounts for community richness, as well as the relative rarity of species present (Chadd and Extence 2004). The CCI method assigns a conservation score to each species based on their relative rarity, averaged across sites and multiplied by a community score, derived from either the rarest taxon present, or the Biological Monitoring Working Party (BMWP) score (Chesters 1980); we used the latter. High CCI scores thus indicate high taxon richness and/or high rarity, reflecting the presence of nationally rare species. Taxon richness, Shannon diversity (Shannon and Weaver 1949) and Pielou (Pielou 1966) evenness scores were also calculated for each pond. Rare species were identified as Red Data Book (1–3), Notable (A or B) or Regionally Notable (NR), sensu Chadd and Extence (2004).

Macroinvertebrate community data were tested for spatial autocorrelation at 500 m intervals within a 12.5 km neighbourhood using a Mantel correlogram in the R package ‘vegan’ (Oksanen et al. 2015). At no distance was the Mantel correlation coefficient significant (999 permutations, Bonferroni corrected).

The R package ‘randomForest’ (Liaw and Wiener 2002) was used to develop a random forest model that predicted cluster membership of the ponds from physicochemical, connectivity and land-use data. The statistical associations between ecological data are frequently non linear and complex, which limit the ability of conventional statistical approaches to provide meaningful analyses (Austin and Meyers 1996; De’ath and Fabricius 2000). To this end machine learning classifiers, such as RF, are increasingly being used for predictive ecological modelling (Finn and Poff 2008; Crisci et al. 2012). In order to aid interpretation, only one of any two highly collinear variable pairs (>0.7 Spearman’s) were retained within the analysis (see Supplementary material T1).

Six preliminary models established the most relevant spatial extent of land-use data using the concentric ring approach (Table 2). After establishing the most relevant spatial extent, values for land-use variables were incorporated into a second, global model combining physicochemical, connectivity and land-use data. In developing the RF models, variables were removed in a stepwise manner according to the mean decrease in accuracy (MDA) scores. MDA is calculated as the normalised difference between ‘out-of-bag’ (OOB) accuracy of the original observations to randomly permuted variables (Cutler et al. 2007). Variables were removed at each model iteration until all variables had positive MDA scores, where negative values indicated that the variable added negligible information to the model (i.e. created noise).

**Table 2:** Out-of-bag (OOB) error estimates for landscape-scale models incorporating concentric ring analysis of land-use factors and a global model combining local with landscape-scale (100 m) factors for predicting classification of four pond types

Model	Formula	Important variables (MDA >0)	OOB (%)
Landscape 50 m	~IS + IG + Scrub + Tree	Scrub (5.88), Tree (5.18), IS (5.07)	66.7
100 m		IS (9.80), Scrub (4.08), Tree (3.10)	53.3
250 m		IS (9.97), Scrub (8.09), IG (0.05)	63.3
500 m		IS (9.78), Scrub (6.30), Tree (4.10), IG (2.62)	63.3
1000 m		n/a	86.7
2500 m		Tree (5.09), Scrub (3.56), IS (3.37)	63.3
Global	~Pond.500 m + IS.100 m + Scrub.100 m + Concrete + Shading + Fringing + Floating + Mphyte + WLFI + K + NH_4_ + Chl-*a*	Mphyte (6.45), IS.100 m (4.10), Shading (3.82), K (2.70), Floating (2.51), Scrub.100 m (1.93), Concrete (1.58), Pond.500 m (1.33) Chl-*a* (1.33), NH_4_ (0.61), Fringing (0.37), WLFI (0.32)	36.7

*IS* impermeable surface, *IG* improved grassland, *Scrub* vegetation between 0 and 3 m, *Tree* Tree canopy cover (over 3 m)

The number of variables randomly sampled as candidates at each decision tree node split was the square root of the number of predictors (Liaw and Wiener 2002). The number of decision trees constructed was 301 and 501 for the spatial extent and global models respectively, after which OOB error estimates were stable. The global model was validated using repeated k-fold cross-validation (5 repeats, 10 folds). This generated average accuracy, Kappa and observed versus predicted (confusion matrix) statistics which indicate the extent of agreement between repeated models (perfect agreement = 1). Direction of predictor effects was verified for the global model by plotting the partial dependencies of responses to individual predictor variables whilst holding the effect of all other variables constant (Friedman 2001; De’ath 2007; Johnstone et al. 2010); see Supplementary material F2–F13.

## Results

A total of 192 taxa from 14 orders were recorded from the 30 ponds across spring and summer 2009; 157 were identified to species level (see Supplementary material T2). The most species-rich orders were Coleoptera (47 species), Hemiptera (31), Trichoptera (24), Gastropoda (17) and Odonata (15). Seventeen of the 30 sites (57%) contained at least one unique taxon. Mean taxa richness across all sites was 46.5 (range 14–87). On average, each site supported 1.33 and 0.53 non-native macroinvertebrate and macrophyte species respectively from a total of three and seven identified during the study (Table 3).

**Table 3:** Non-native macrophyte and macroinvertebrate species found in each pond type

Order	Family	Species	Type 1	Type 2	Type 3	Type 4
Invertebrates						
Amphipoda	Crangonyctidae	*Crangonyx pseudogracilis*	10 (100)	5 (72)	7 (100)	5 (83)
Gastropoda	Hydrobiidae	*Potamopyrgus antipodarum*	7 (70)	3 (43)	1 (14)	1 (17)
Gastropoda	Planariidae	*Planaria torva*	0 (0)	0 (0)	1 (14)	0 (0)
Macrophytes						
Saxifragales	Crassulaceae	*Crassula helmsii*	3 (30)	0 (0)	0 (0)	0 (0)
Apiales	Apiaceae	*Hydrocotyle ranunculoides*	1 (10)	0 (0)	0 (0)	0 (0)
Alismatales	Hydrocharitaceae	*Elodea canadensis*	4 (40)	0 (0)	2 (29)	0 (0)
Alismatales	Hydrocharitaceae	*Elodea nuttallii*	2 (20)	0 (0)	0 (0)	0 (0)
Alismatales	Hydrocharitaceae	*Lagarosiphon major*	1 (10)	0 (0)	1 (14)	0 (0)
Saxifragales	Haloragaceae	*Myriophyllum aquaticum*	0 (0)	0 (0)	0 (0)	1 (17)
Salviniales	Azollaceae	*Azolla filiculoides*	0 (0)	0 (0)	1 (14)	0 (0)

The number (and percentage) of sites that supported the species within each pond type are reported

Cluster analysis of macroinvertebrate abundance data identified four groups of ponds, termed Type 1 (*n* = 10 pond sites), Type 2 (*n* = 7), Type 3 (*n* = 7) and Type 4 (*n* = 6) ponds. ANOSIM revealed that differences in structure of these assemblages among the pond types was statistically significant (R = 0.67, *P* < 0.001), although some species occurred in more than one type of pond (see Supplementary material F1). IndVal analysis identified indicator species for three of the four pond types (Table 4). Of the 192 different taxa recorded, 40 were significant indicators (*P* < 0.05) for Type 1 ponds, four for Type 3 and seven for Type 4 ponds, whereas no significant indicators were identified for Type 2 ponds.

**Table 4:** Taxa that most characterised the macroinvertebrate assemblages of pond types as identified but indicator value (IndVal) analysis

Pond type	No. of sites	Top indicator taxa with indicator value (IV), P = < 0.05	Total indicators
1	10	*Phryganea bipunctata* (0.80), *Ischnura elegans* (0.73), *Sympetrum sanguineum (*0.70), *Enochrus testaceus* (0.70), *Lymnaea stagnalis* (0.68), *Noterus clavicornis* (0.68), Anisoptera (instar I -II) (0.67), Zygoptera (instar I-II) (0.66), *Agraylea multipunctata* (0.62), *Gyraulus albus* (0.62)	40
2	7	None identified	0
3	7	*Planorbarius corneus* (0.53), *Radix peregra* (0.49), *Agabus bipustulatus* (0.37), *Crangonyx pseudogracilis* (0.34)	4
4	6	*Hesperocorixa sahlbergi* (0.56), Chaoboridae (0.52), *Colymbetes fuscus* (0.43), *Acilius sulcatus* (0.38), *Corixa punctata* (0.38), *Helobdella stagnalis* (0.37), Corixidae (nymphs) (0.37)	7

### Contrasts among pond types

Type 1 ponds were macrophyte rich with high floating and fringing vegetation cover, unshaded and nutrient poor (PO_4_ and K). Few impermeable surfaces surrounded them, with relatively high levels of scrub and connectivity to other wetlands in the wider environment (Table 5). The macroinvertebrate communities of Type 1 ponds were of high conservation value (mean CCI = 12.4) and the most taxonomically rich sites surveyed (see descriptors, Table 6). The IndVal analysis identified 40 taxa from ten taxonomic orders as indicators across the four pond types. Indicators of Type 1 ponds included ten caddisflies (Trichoptera), with *Phryganea bipunctata* and *Agraylea multipunctata* being the strongest indicator taxa in this group (Table 4). Other indicators included eight dragonflies (Odonata), especially *Ischnura elegans* (Zygoptera: Coenagriidae) and *Sympetrum sanguineum* (Anisoptera: Libellulidae). Of the remaining indicator taxa, two water beetles *Enochrus testaceus* (Coleoptera: Hydrophilidae) and *Noterus clavicornis* (Coleoptera: Noteridae), the snail *Gyraulus albus* (Gastropoda: Planorbidae) and true bugs *Ranatra linearis* (Hemiptera: Nepidae) and *Ilycoris sp*. (Hemiptera: Naucoridae) were indicators of Type 1 ponds (range 0.6–0.7, *P* < 0.01). The high number of significant indicators reflects that 55 taxa were recorded exclusively in Type 1 ponds (Table 6), and of these 23 were recorded at only one single site (site 21).

**Table 5:** Mean ± 1SD (min–max) values of physicochemical and land-use variables within clustered pond types identified as important predictors of group classification

	Type 1 (Macrophyte-rich, open, low nutrients)	Type 2 (Concrete, macrophyte-poor, high nutrients, urban)	Type 3 (Macrophyte-rich, low nutrients, ephemeral)	Type 4 (Shaded, macrophyte-poor, high nutrients, urban)
Local factors				
Macrophyte taxa	11.8 ± 4.1 (7–20)^a^	1.7 ± 1.7 (0–4)^b^	8.6 ± 4.7 (3–15)^ac^	3.7 ± 1.8 (1–6)^bc^
Phosphate (mg/L)	0.1 ± 0.09 (0.0–0.3)^a^	1.0 ± 0.76 (0.2–2.1)^b^	0.2 ± 0.12 (0.1–0.4)^ab^	0.9 ± 0.85 (0.1–2.5)^b^
Shading (%)	5.9 ± 7.2 (0.0–16.7)^a^	40.9 ± 20.0 (19.1–69.3)^b^	31.1 ± 38.1 (2.6–95.0)^ab^	56.9 ± 34.6 (6.3–100)^b^
Potassium (mg/L)	4.1 ± 1.5 (1.5–7.7)^a^	4.3 ± 0.8 (2.9–5.1)^ab^	4.4 ± 2.8 (1.6–9.6)^ab^	7.0 ± 2.9 (4.7–12.4)^b^
Floating cover (%)	11.6 ± 13.9 (0.5–47.2)^a^	1.2 ± 1.1 (0.0–3.2)^ab^	2.6 ± 6.1 (0.0–16.4)^b^	1.3 ± 3.0 (0.0–7.4)^b^
Concrete^†^	2.8 ± 8.1 (0.0–25.9)^a^	37.1 ± 34.3 (0.0–100)^b^	14.6 ± 14.3 (0.0–36.6)^ab^	8.5 ± 8.3 (0.0–18.9)^ab^
Chl *a* (µg/l)	32.4 ± 19.4 (10.2–73.6)	35.2 ± 28.0 (9.5–74.2)	72.5 ± 81.1 (4.0–243.7)	156.9 ± 159.0 (8.4–437.9)
Ammonia (mg/L)	0.2 ± 0.1 (0.0–0.3)	0.9 ± 1.1 (0.0–2.7)	0.9 ± 2.1 (0.0–5.7)	1.5 ± 1.1 (0.1–3.2)
Fringing cover (%)	18.0 ± 15.6 (0.2–37.3)	7.0 ± 2.5 (3.1–10.1)	17.1 ± 16.6 (0.0–41.4)	5.0 ± 6.4 (0–16.1)
WLFI^*^	99.8 ± 107 (4.1–353.6)	27.5 ± 21.9 (6.4–69.8)	1250.1 ± 1995 (12.0–4698)	64.2 ± 48.6 (20.4–126.9)
Landscape factors (% cover)				
Impermeable surfaces (100 m)	53.2 ± 7.7 (23.6–82.6)^a^	66.6 ± 5.7 (58.5–78.9)^b^	68.9 ± 9.8 (48.2–91.7)^ab^	66.3 ± 7.8 (43.3–90.2)^b^
Scrub <3 m vegetation (100 m)	27.0 ± 15.0 (14.4–44.8)^a^	16.2 ± 2.6 (11.6–21.0)^ab^	19.5 ± 10.6 (10.2–25.4)^ab^	15.1 ± 5.4 (10.7–21.2)^b^
Ponds % (500 m)	9.5 ± 5.2 (2.0–15.2)^a^	2.1 ± 2.3 (0.0–6.4)^b^	6.1 ± 6.3 (0.0–17.9)^ab^	2.6 ± 2.2 (0.0–5.0)^ab^
Water % (500 m)	20.4 ± 7.4 (9.9–31.5)^a^	7.1 ± 3.2 (3.6–12.9)^b^	15.1 ± 4.7 (10.2–24.7)^ab^	6.1 ± 2.8 (2.5–11.0)^b^

Lettering denotes significant differences between pond types (Kruskal–Wallis, post hoc Dunn, *P* < 0.05 Bonferroni corrected)

^*^Wetland Level Fluctuation Index–the standard deviation of three measures of water depth at fixed points (measured Spring, Summer and Autumn)

^†^Percentage of bank formed of hard engineering e.g. concrete

**Table 6:** Mean ± 1SD (min–max) Community Conservation Index score, taxa richness, Shannon diversity index and Pielou measure of evenness within each pond type (italicised are values without non-native species) and the total number of taxa, unique taxa (exclusive to that pond type) and rare (see Supplementary material T3) species occurring across each pond type

	Type 1	Type 2	Type 3	Type 4
CCI^a^	16.9 ± 3.6 (11.1–26.4)	8.9 ± 5.4 (4.6–19.8)	10.3 ± 0.87 (8.8–11.3)	8.8 ± 5.4 (1.4–17.0)
	*17.22* ± *3.7 (11.3*–*25.9)*	*9.3* ± *5.7 (4.6*–*21.5)*	*10.3* ± *0.71 (9.0*–*10.9)*	*9.1* ± *5.4 (1.4*–*17.5)*
Taxon richness	69 ± 10.6 (54–87)	23 ± 9.3 (14–39)	47 ± 9.27 (32–59)	37 ± 11.3 (18–51)
	*67* ± *10.5 (52*–*85)*	*22* ± *8.7 (14*–*37)*	*46* ± *9.41 (30*–*58)*	*36* ± *11.1 (18*–*50)*
Shannon	2.59 ± 0.39 (1.82–3.02)	1.39 ± 0.54 (0.60–2.33)	1.87 ± 0.55 (1.15–2.60)	1.73 ± 0.27 (1.53–2.25)
	*2.54* ± *0.38 (1.78*–*2.95)*	*1.32* ± *0.51 (0.60*–*2.25)*	*1.75* ± *0.59 (0.89*–*2.52)*	*1.64* ± *0.30 (1.28*–*2.16)*
Pielou	0.61 ± 0.09 (0.44–0.67)	0.45 ± 0.15 (0.23–0.67)	0.49 ± 0.14 (0.31–0.67)	0.49 ± 0.05 (0.44–0.57)
	*0.60* ± *0.08 (0.43*–*0.69)*	*0.43* ± *0.14 (0.23*–*0.66)*	*0.46* ± *0.15 (0.24*–*0.66)*	*0.46* ± *0.07 (0.38*–*0.55)*
Total unique taxa	55	3	9	3
Total rare species	6	2	2	2
Total taxon richness	167	73	122	89

^a^Community Conservation Index (after Chadd and Extence 2004)

Type 1 ponds contained the most rare and notable taxa (6, see Supplementary material T3) including the regionally notable *Limnephilus decipiens* (Trichoptera: Limnephilidae) and nationally scarce *Helochares lividus* (Coleoptera: Hydrophilidae). 70% of Type 1 ponds contained fish and the leech *Piscicola geometra* (Hirudinea: Piscicolidae)–a sanguivorous ectoparasite of freshwater fish in well oxygenated waters (Elliott and Mann 1998). Type 1 ponds most regularly supported non-native plant and macroinvertebrate species (Table 3) including the highly invasive New Zealand Pygmyweed (*Crassula helmsii*) and floating pennywort (*Hydrocotyle ranunculoides*).

Type 2 ponds were found in highly urbanised surroundings with few wetlands in the wider landscape. Type 2 ponds had hard-engineered banks, and high phosphate concentrations. These shaded sites (by overhanging trees) contained fewer macrophytes (mean 1.7 taxa) than other ponds (Table 5). Macroinvertebrate diversity and evenness was also degraded, being lowest of any pond type (Table 6). On average, Type 2 sites had CCI scores that were of moderate conservation value (Chadd and Extence 2004). Nevertheless a minority of sites in the group Type 2 supported communities of higher conservation value (CCI 19.8, site 7) reflecting the occurrence of rare, Notable B species *Hydroglyphus geminu*s (Coleoptera: Dytiscidae) which is often found in sites with fluctuating margins and silt (Foster and Friday 2011), and Notable Regional taxon *Micronecta scholtzi* (Hemiptera: Micronectidae) characteristic of ponds and lakes with bare mineral (e.g. gravel) bottoms. There were no indicator species for Type 2 ponds, which contained mainly cosmopolitan, pollution tolerant taxa, especially Chironomidae, *Asellus aquaticus* (Amphipoda: Asellidae) and Oligochaeta (totalling up to 81% of total numbers). No non-native macrophyte and macroinvertebrate taxa were collected from Type 2 ponds (Table 3).

Type 3 ponds were the least well defined. They tended to have good macrophyte richness and high floating and fringing vegetation cover with the most fluctuating water levels of any pond type. Type 3 ponds were generally in less urban, and more naturalised (scrubby) settings (Fig. 3; Table 5). The ponds were of moderate-high conservation value (CCI range 9.0–11.3) and both macroinvertebrate taxa richness (mean 47 taxa) and Shannon diversity (mean 1.87) were the second highest of any pond type (Table 6).

The IndVal analysis identified four indicator species of Type 3 ponds (Table 4), namely *Planorbarius corneus* (Gastropoda: Planorbidae) present at six from the seven ponds, with *Radix peregra* (Gastropoda: Lymnaeidae) a moderate indicator. Nine taxa were found exclusively in Type 3 ponds (Table 6): five coleoptera, two odonata, *Stagnicola palustris* (Gastropoda: Lymnaeidae) and *Planaria torva* (Tricladida: Planariidae). The introduced shrimp *Crangonyx pseudogracilis* (Amphipoda: Crangonyctidae) was a weak but significant indicator and present in all Type 3 ponds, whilst both native Gammaridae recorded elsewhere during the study were absent. Type 3 ponds supported several non-native macrophyte and macroinvertebrate species (Table 3) including the invasive water fern (*Azolla filiculoides*).

Type 4 were the most urbanised and shaded, nutrient (PO_4_ and K) rich sites frequently dominated by phytoplankton (Chl-*a* 8.4–437.9 µg/L) and with few macrophytes (Table 5). The conservation value of these sites varied from moderate-high (CCI 5.1 to 17) but taxon richness and evenness were moderate to low (Table 6). The IndVal analysis identified seven indicator species, of which *Hesperocorixa sahlbergi* (Hemiptera: Corixidae) and the family Chaoboridae (Diptera) were the strongest indicators. Chaoboridae, which are frequently found in greater abundance in the absence of fish (Schilling et al. 2009) were present at the six fishless Type 4 ponds, and sometimes dominated the macroinvertebrate community (33% of total numbers at site 11). As a detritus feeder (Tachet et al. 2002) *H. sahlbergi* is frequently found in water bodies rich in organic matter. Of the remainder, *Colymbetes fuscus* (Coleoptera: Dytiscidae) was a moderate indicator and *Acilius sulcatus* (Coleoptera: Dytiscidae), *Corixa punctata* (Hemiptera: Corixidae), *Helobdella stagnalis* (Hirudinea: Glossiphoniidae) and Corixidae nymphs were all weak but significant. A single private ornamental Type 4 pond (site 19) supported the non-native and invasive parrot’s feather (*Myriophyllum aquaticum*).

### Influential factors on pond classification

A global random forest model correctly predicted pond type for 19 of the 30 ponds (62.7% accuracy) from physicochemical, land-use and connectivity data. A Kappa statistic of 47.3 suggested a good degree of agreement between resampled models (Landis and Koch 1977) and an OOB error estimate of 36.7 considerably improved upon the concentric ring analysis incorporating land-use only (Table 2). On average the model performed very well for Type 1 (96.7% of pond membership predictions correct) and Type 2 ponds (71.6%), moderately for Type 4 ponds (39%), but performed poorly for Type 3 ponds (24%).

Six initial random forest models were used to identify the most relevant spatial extent from the pond edge for land-use variables. From these the OOB error estimates ranged between 53.3 and 86.7%, with the best model using land-use data (excluding improved grassland) from 100 m to the pond edge (Table 2). Thus, land-use variables within 100 m were entered into the global model to be contrasted against local factors and connectivity indices.

**Fig. 2:**
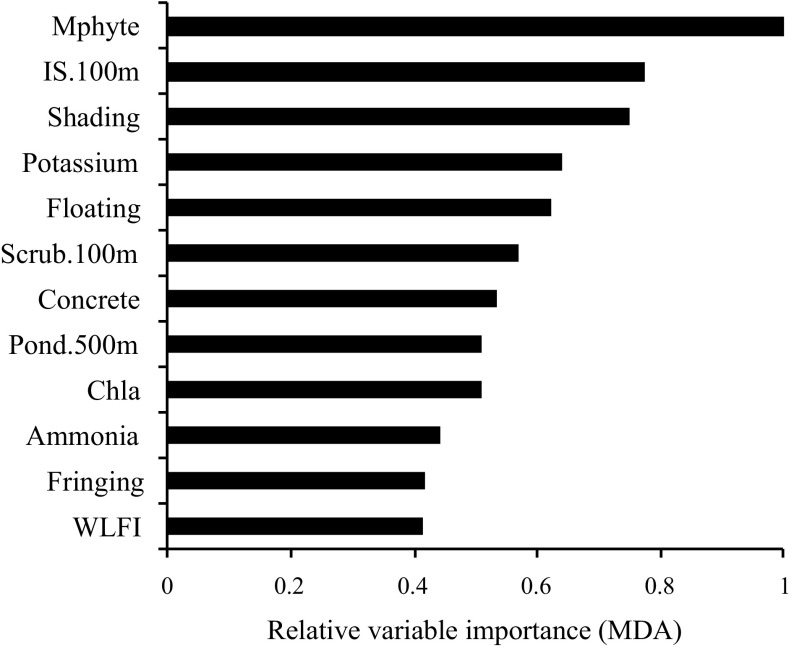
Influential local and landscape-scale variables used by a global random forest model to predict pond type membership. The greater the value of the mean decrease in accuracy (MDA) statistic the greater the loss of model predictive accuracy when that variable is excluded (or permuted) from decision trees

**Fig. 3:**
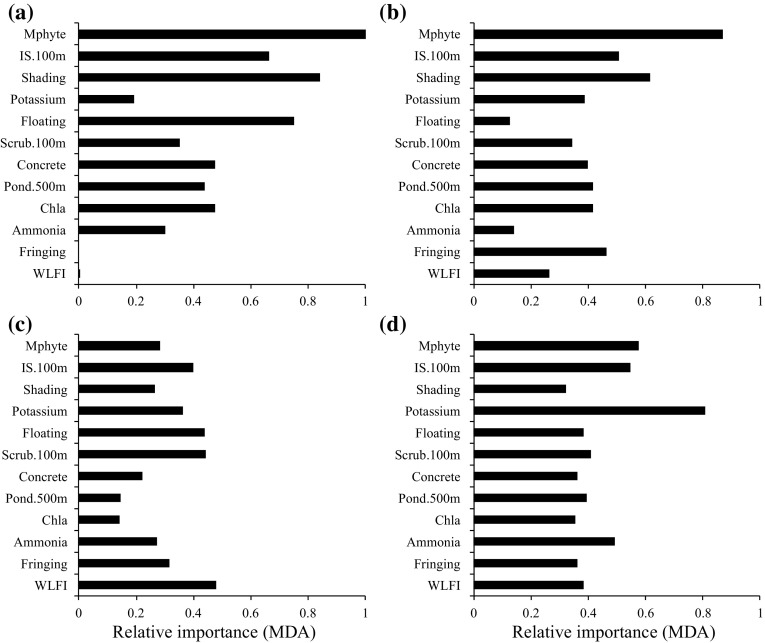
Influential local and landscape-scale variables used by a global random forest model to predict pond type membership broken down into pond types, **a** Type 1, **b** Type 2, **c** Type 3 and **d** Type 4. The greater the value of the mean decrease in accuracy (MDA) statistic the more relevant the variable is for classification into the pond type

Macrophyte richness was the most important predictor of pond type in the global model, as indicated by the mean decrease in accuracy (MDA) statistic (Fig. 2 and Fig. 3). Highly collinear (ρ −0.76, *P* < 0.05) with macrophyte richness was phosphate concentration (range 0.02–2.46 mg/L) and the abundance of wetlands within 500 m (ρ 0.73, *P* < 0.05). The degree of riparian shade (range 30–100%) was of secondary importance, with a similar influence to the proportion of impermeable surfaces within 100 m i.e. the most influential landscape-scale factor (Fig. 2). Dissolved potassium concentration, floating vegetation cover, proportion of hard engineering along the pond perimeter (range 0–1), the abundance of ponds within 500 m and coverage of scrub within 100 m were moderately influential. Chlorophyll *a* concentration, the amount of fringing vegetation cover, water level fluctuation (WLFI) and ammonia concentration were also important, but did not significantly differ between pond types (Table 5).

## Discussion

Our study of 30 ponds across an urban land-use gradient in the West Midlands, UK, revealed important associations among local physicochemical factors, surrounding land-use and macroinvertebrate assemblage composition. Most notably, we identified key pond types associated with repeating patterns of nutrient status, the degree of riparian shading and extent of hard engineering that influenced the structure of macroinvertebrate assemblages and their conservation value. Random forest models indicated that it was the land-use immediately surrounding ponds (within 100 m) that exerted the strongest influence on pond biota (Table 2), and although local factors often had primacy, ponds in the most highly urbanised surroundings were often of the lowest conservation value.

The ponds varied markedly in taxon richness (range 17–82 taxa) and the median (48) was higher than that reported in pond surveys in both urban (Gledhill et al. 2008; Vermonden et al. 2009; Noble and Hassall 2014; Hill et al. 2015) and rural settings (Williams et al. 2004); although differences in sampling methods and taxonomy are acknowledged. Within our dataset, ponds rich in macrophytes (i.e. Types 1 and 3) supported the most taxonomically diverse macroinvertebrate assemblages, and were often rich in Coleoptera, Hemiptera, Gastropoda and Trichoptera. Macrophyte diversity is well known to improve macroinvertebrate habitat complexity by providing food and refuge from predation (Gilinsky 1984; Williams 1997) and positive relationships between macroinvertebrate and macrophyte richness have been observed elsewhere (Gledhill et al. 2008; Hassall et al. 2011; Hill et al. 2015). Macrophyte stands provide habitat for a wide range of macroinvertebrates that are preyed on by odonates (Lombardo 1997) and emergent vegetation provides resting and mating sites for adult dragonflies and damselflies (Remsburg et al. 2008). Cased caddis require plant material to build their cases and caseless forms are often found attached to macrophytes (Samways and Steytler 1996; Schindler et al. 2008). They also serve as a substrate for epiphytic algae that provide food for herbivorous gastropods (Bronmark 1985) and provide refuge from predation by molluscivorous fish. Our data suggest macroinvertebrate assemblages in ponds lacking macrophytes tend to be relatively impoverished.

Ponds with heavy riparian shading and hard-engineering were macrophyte-poor and turbid (i.e. Type 2 ponds). Deep shade (e.g. >75% tree cover) can increase nutrient concentrations in some ponds, both directly via leaching from abscissed leaves (Adámek and Maršálek 2013), and indirectly as the constrained photosynthetic capacity of submerged plants limits nutrient uptake from the water column (Jeppesen et al. 1997). However, there is some evidence that moderate shading can benefit the diversity of pond macrophytes (Biggs et al. 1994; Gee et al. 1997). For instance, Gee et al. (1997) reported maximum macrophyte species richness in ponds with approximately 30% riparian shade, a pattern not dissimilar to that seen in ponds of Type 3 here. Although the mechanistic basis for the relationship is unclear, it has been suggested that shading moderates extremes of water temperature (Gee et al. 1997) and/or prevents dominance by fast growing macrophyte species (Dawson and Haslam 1983). Some shading may also provide protection against wind, which might otherwise increase wave action, re-suspend sediment and stimulate nutrient turnover.

Consistent with other research, we found that regional aquatic habitat availability (lentic or lotic systems) was a strong determinant of macrophyte species richness (Gledhill et al. 2008; Akasaka et al. 2010). The availability of pond habitat only was a weaker correlate with macrophytes, however the association was important within the global random forest model nevertheless. Thus, it is likely that the availability of a range of aquatic water bodies representing a multitude of environmental conditions is important in sustaining the diversity of ecological elements in ponds (i.e. macrophytes and macroinvertebrates), some of which are pond specialists and others which are more cosmopolitan (Williams et al. 2004).

Most of the study ponds in the West Midlands were nutrient rich, with phosphate concentrations (26 ponds >0.031 mg/L, geometric mean) indicative of eutrophic conditions in shallow lakes (UK TAG, 2008) and being comparable to old industrial mill ponds (Wood and Barker 2000) and in the same order as ponds within agriculturally intense landscapes (Williams et al. 2004). Potassium was also particularly high in Type 4 sites, suggestive of NPK fertilizer use in local catchments (Talling 2010). Eutrophication can occur as a result of natural processes or pollution and can reduce dissolved oxygen concentrations, especially in summer (Gee et al. 1997; Birch and Mccaskie 1999; Angélibert et al. 2004). Oxygen depletion was relatively common in our study ponds, with about one third of sites suffering oxygen sags below 20% saturation (Thornhill 2013). In this respect, our urban sites appear to be more impacted than some nutrient-rich rural ponds, where oxygen depletion is less frequent (Williams et al. 2004). The disparity may be explained by a relative dearth of macrophytes in our sites (this study: mean 7.1 taxa; Williams et al. 2004: mean 10.1 taxa), with more rural ponds existing in a clear-water state sustained by more complex macrophyte assemblages (Scheffer et al. 1993).

Concentric ring analysis revealed that the pond assemblages were influenced by land-use surrounding the ponds, especially the degree of urbanisation and the presence of naturalised land within 100 m of the pond edge. This finding is consistent with studies that show how surrounding land-use influences pond water quality (Declerck et al. 2006; Akasaka et al. 2010) and macrophyte richness (Williams et al. 2010; Akasaka et al. 2010). Urban activities cause point-source and diffuse pollution (Faulkner et al. 2000) and result in the direct mortality of biota (Kriska et al. 1998). Urban landscapes also present major obstacles to dispersal, such as light pollution (Bilton et al. 2001; Horváth et al. 2007) and road infrastructure (e.g. amphibians, Parris 2006; caddisflies, Blakely and Harding 2005). The link between biota in urban ponds and their immediate surroundings may reflect the absence of vegetated buffer strips which might otherwise intercept chemicals from surface water run-off such as fertilisers and herbicides (Sliva and Williams 2001).

We found contrasting pond types in the West Midlands area, influenced to differing extents by a range of local and landscape-scale factors. Ponds are discrete habitats with small catchments (Davies et al. 2007) rooted in spatially complex landscapes. Localised conditions generated diverse physical and chemical conditions, and hence ecological niches, across the pond network (Biggs et al. 2005). Stressors have had marked impacts on local species complements, but do not propagate to other ponds as readily as occurs upstream–downstream in lotic systems. This combined patchiness and isolation of pond communities may thus confer resilience to the metapopulations of pond networks, maintaining regional biodiversity despite locally deleterious conditions.

Despite the heavily altered and urban landscape of Birmingham and Black Country, a number of ponds of moderate-high conservation value (i.e. CCI scores of >10) were identified. Scores in this range are typical of sites supporting at least one uncommon species, or several species of restricted distribution, or a community with high taxon richness. We recorded one Red Data Book species (RDB3–Nationally rare (Hyman and Parsons 1992)), four Notable B species and three Notable Regional species (see Supplementary material T3). Only two ponds were of low conservation value (CCI < 5), being species poor or containing only common species (Chadd and Extence 2004). However, only one pond (Site 21, within a protected wetland) was classed as being of very high conservation value (CCI score of 26.4). The pond was taxonomically diverse and contained the only RDB3 listed species–*Hydrochus elongatus*–found during the study. *H. elongatus* is a water scavenger beetle characteristic of ponds and drains and often is found in reed beds.

Across the study a third of ponds contained at least one non-native invasive macrophyte species as listed in Schedule 9 of the UK Wildlife and Countryside Act (1981), with non-native species presence associated with higher macroinvertebrate taxa richness and Shannon diversity (Mann–Whitney, *P* < 0.05). Whilst this does not imply a causal link, the finding is consistent with the Countryside Survey 2007 (Williams et al. 2010) which found that the prevalence of non-native species was associated with higher macrophyte diversity. In addition, the non-native amphipod *C. pseudogracilis* was present in 87% of ponds in this study and in 77% of the most highly impacted Type 2 and 4 ponds where native amphipods of the family Gammaridae were absent in 92% of cases. This finding reflects those of several authors who have suggested that due to an ability to tolerate poor physicochemical conditions *Crangonyx* spp. have the potential to fill the niche left vacant by native *Gammarus* spp. (MacNeil et al. 1999; Vermonden et al. 2009). Nevertheless, invasive non-native species could threaten the ecology of urban areas (Shochat et al. 2010) including ponds (Wood and Barker 2000; Williams et al. 2007). The full impact of non-native species can take time to develop (Strayer et al. 2006) and the historical presence of non-native species across the study sites is not known. However, this study suggests that the presence of some non-native species in stressed environments such as urban ponds does not obviate a negative impact and requires further research (Stendera et al. 2012).

Fish were present in 13 (43%) of the study ponds, of which 11 were either Type 1 (high conservation value) or 2 ponds (low conservation value). Fish are known to reduce macroinvertebrate species richness in ponds and lakes (e.g. Wood et al. 2001; Schilling et al. 2009) but we found no consistent evidence for that here. In highly modified systems, fishery management could offset potentially negative impacts to biodiversity as it often aims to keep selected areas of open water and remove overhanging vegetation to improve fishermen access as well as remove fine sediment to maintain water depth (Linton and Goulder 2000; Wood et al. 2001). Alternatively, the relative impacts of fish species composition and stocking densities need further consideration within these sites.

## Management implications

This study identifies the potential for urban ponds to support diverse ecological communities of high conservation value, however many are in an impoverished state. Ponds are small habitats that are both vulnerable to environmental change and tractable as management option such that small changes can have a big impact. In addition, many ponds are in managed public spaces and golf courses (e.g. Jeffries 2012) such that improved management action is the necessary step. Conversely, sites outside of public spaces are likely neglected, with increases in tree cover, lower macrophyte diversity and ultimately lost to sedimentation. This study indicated that local physical factors had a greater combined influence on the macroinvertebrate assemblages than those at a landscape-scale and thus, efforts should first focus on the local pond habitat before land-use within 100 m of the pond edge in order to improve biodiversity. Accordingly, we offer the following priority aims as guidance for improving the conservation value of urban ponds:

- Aim 1: Improve conditions for macrophyte growth

Our data suggest that macrophytes are key biological elements of urban ponds that are important for habitats to sustain high levels of macroinvertebrate biodiversity. Management should encourage growth of macrophytes by reducing shading by tree cover to less than 50% to allow greater light penetration to the water’s surface whilst retaining habitat heterogeneity offered by tree cover. To maximise the benefits of tree-shading reduction, opportunities should be sought to replace hard engineering with marginal habitat of a shallow gradient that maximises the drawdown zone, thus providing a variable depth suitable for a range of macrophyte species to establish.

- Aim 2: Reduce nutrient inputs

Nutrient concentrations were strong negative correlates of macrophyte richness. Across 30 sites, few had stream inflows with most receiving run-off from the surrounding land or built environment where the highest nutrient concentrations were found in the most urban settings. To this end management should seek to reduce household drainage misconnections and increase natural filtration of surface run-off (see Aim 3). Reduction in shading (Aim 1) may also reduce nutrient input derived from allochthonous inputs such as leaf litter.

- Aim 3: Assess options within 100 m to allow naturalised vegetation to develop

Sites that had a higher cover of scrub (vegetation between 0 m and 3 m) within 100 m tended to contain biota of higher conservation value. Management of planting around ponds could help limit polluting runoff from impermeable surfaces (e.g. Type 3 ponds) and naturalised vegetation around ponds is also likely to benefit aquatic insects in their terrestrial phases, offering foraging opportunities and protection from predators, as well as supporting terrestrial fauna and flora.

- Aim 4: Plan on a landscape-scale

Individual site managers need to collaborate in order to promote more resilient pond networks to act as stepping stones for the dispersal of ecological communities, and to improve recolonisation potential should individual ponds suffer pollution impacts. In so doing, pond networks should be comprised of a variety of habitats, recognising that even sites of low conservation value can support species that are unique within the regional species pool.

## Electronic supplementary material

Below is the link to the electronic supplementary material.

Supplementary material 1 (DOCX 1076 kb)
