# Supplementary material for: Local and landscape scale determinants of macroinvertebrate assemblages and their conservation value in ponds across an urban land-use gradient
Source: Biodivers Conserv. 2017 Jan 17;26(5):1065–86. doi: 10.1007/s10531-016-1286-4 (PMC7010385; doi:10.1007/s10531-016-1286-4)
Supplement: Supplementary file 1 — Supplementary material 1 (DOCX 1076 kb) [file 10531_2016_1286_MOESM1_ESM.docx]

# Local and landscape scale determinants of macroinvertebrate assemblages and their conservation value in ponds across an urban land-use gradient

Ian Thornhill^1^, Lesley Batty^1^, Russell G. Death^2^, Nikolai R. Friberg^3,4^, Mark E. Ledger^1^

^1^School of Geography, Earth and Environmental Sciences, University of Birmingham, Edgbaston, Birmingham, West Midlands, B15 2TT, United Kingdom*

^2^ Institute of Agriculture and Environment - Ecology, Private Bag 11-222, Palmerston North 4442, New Zealand.

^3^ Norwegian Institute for Water Research (NIVA), Gaustadalléen 21, 0349 Oslo, Norway

^4^ water@leeds, School of Geography, University of Leeds, Leeds LS2 9JT, United Kingdom

*Corresponding author (ian.thornhill@live.co.uk, +044(0)7860618575)

**Supplementary material**


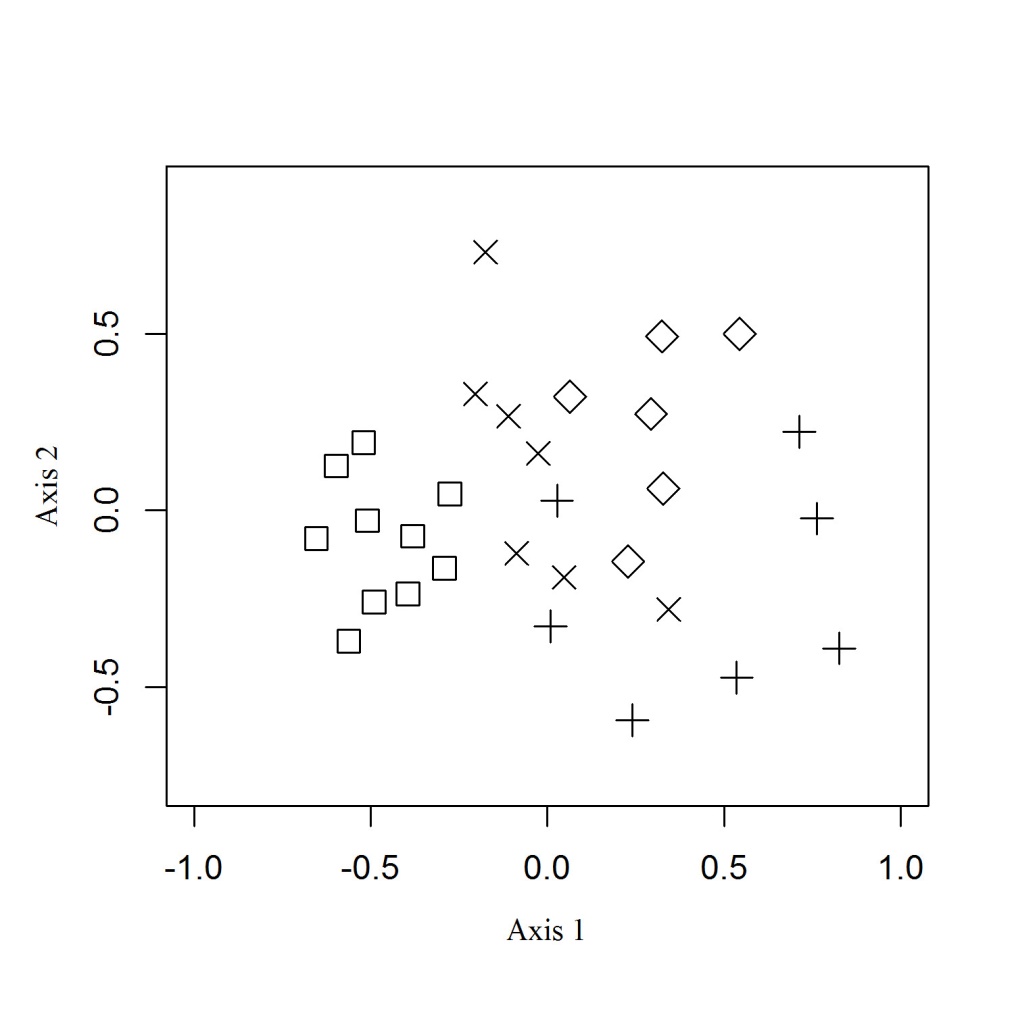


F1. Two-dimensional NMDS plot of dissimilarity (Bray–Curtis index) of macroinvertebrate communities in four types of pond. Type 1 = □, Type 2 = +, Type 3 = ×, Type 4 = ◊ and dots show taxon distribution. Two-dimensional stress = 0.199. Pond types significantly different (ANOSIM, R = 0.67, P < 0.001)


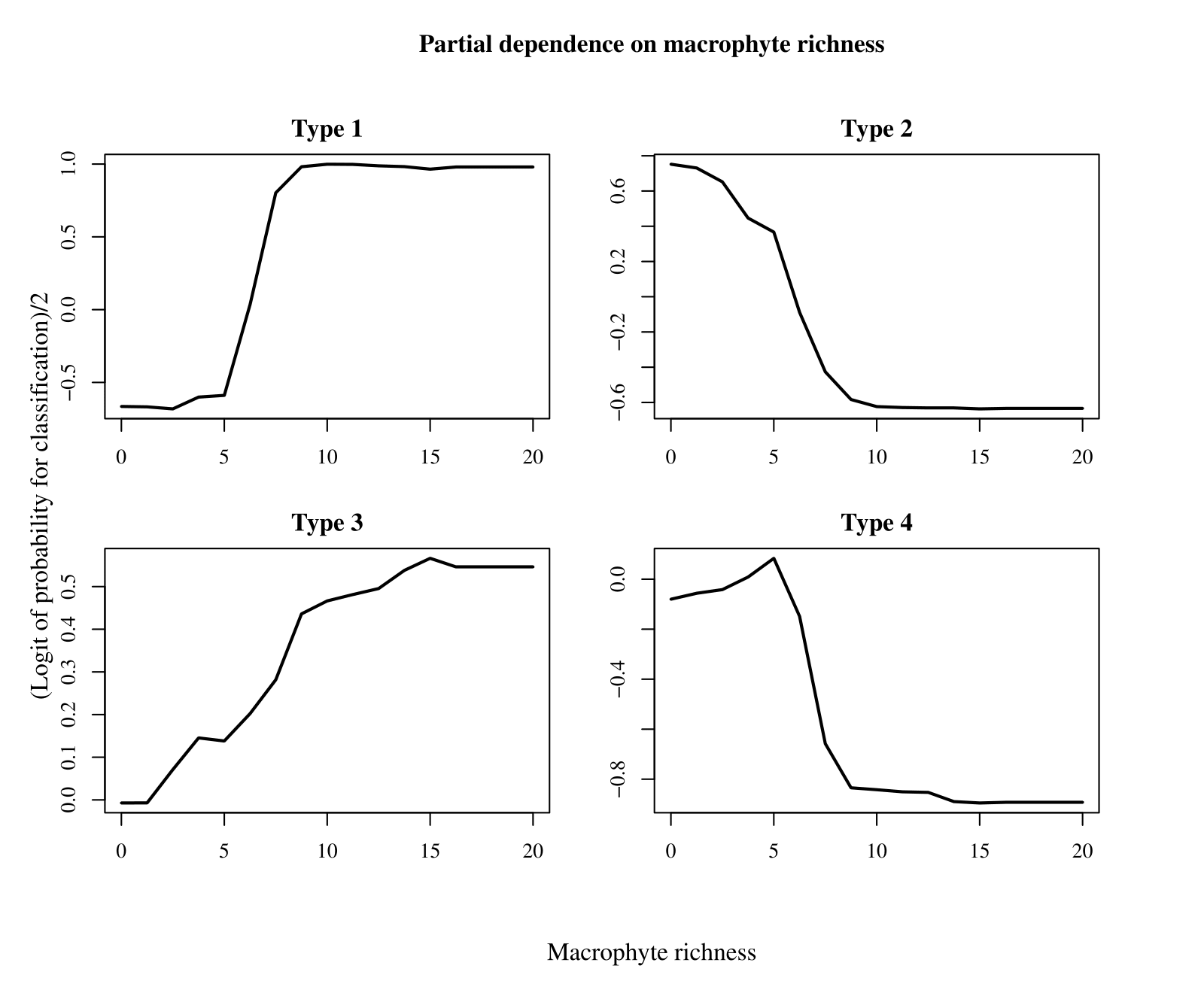
F2 The probability of a pond being classified as Type 1 or 3 is increased with macrophyte richness.


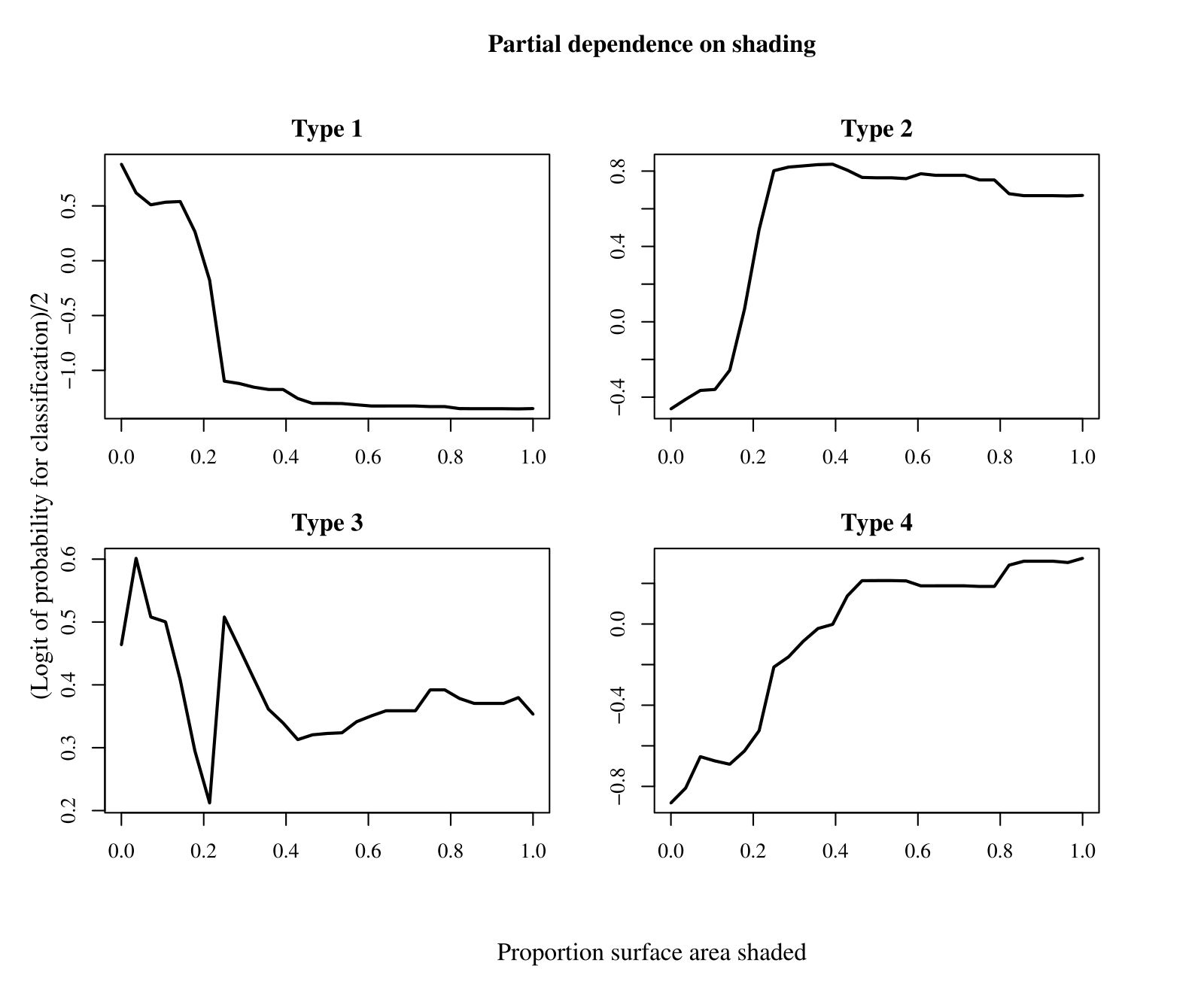
F3 The probability of a pond being classified as Type 2 or 4 increases with more shading, the opposite effect is shown for Type 1


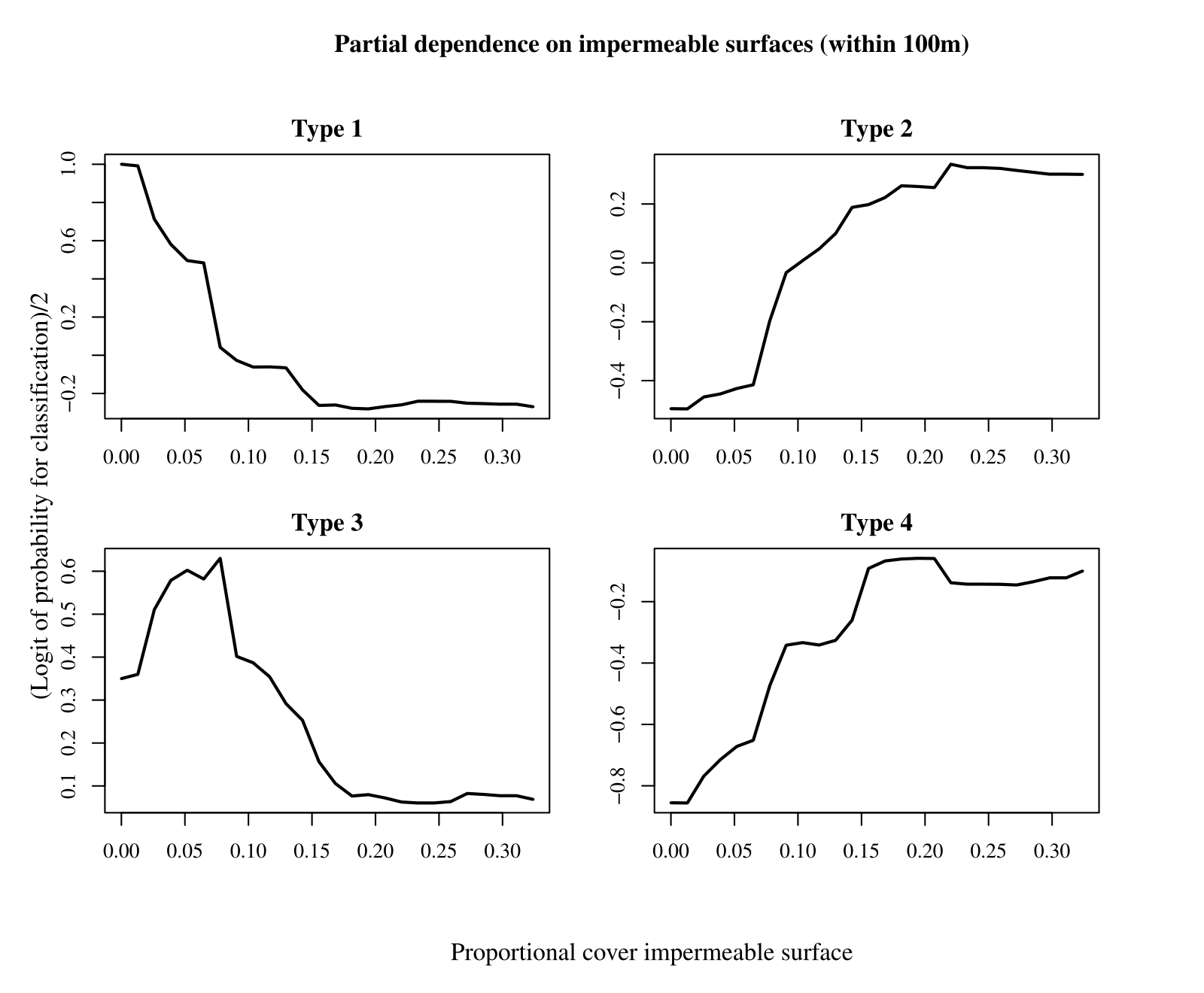
F4 The probability of a pond being classified as Type 2 or 4 increases with increasing impermeable surfaces within 100m


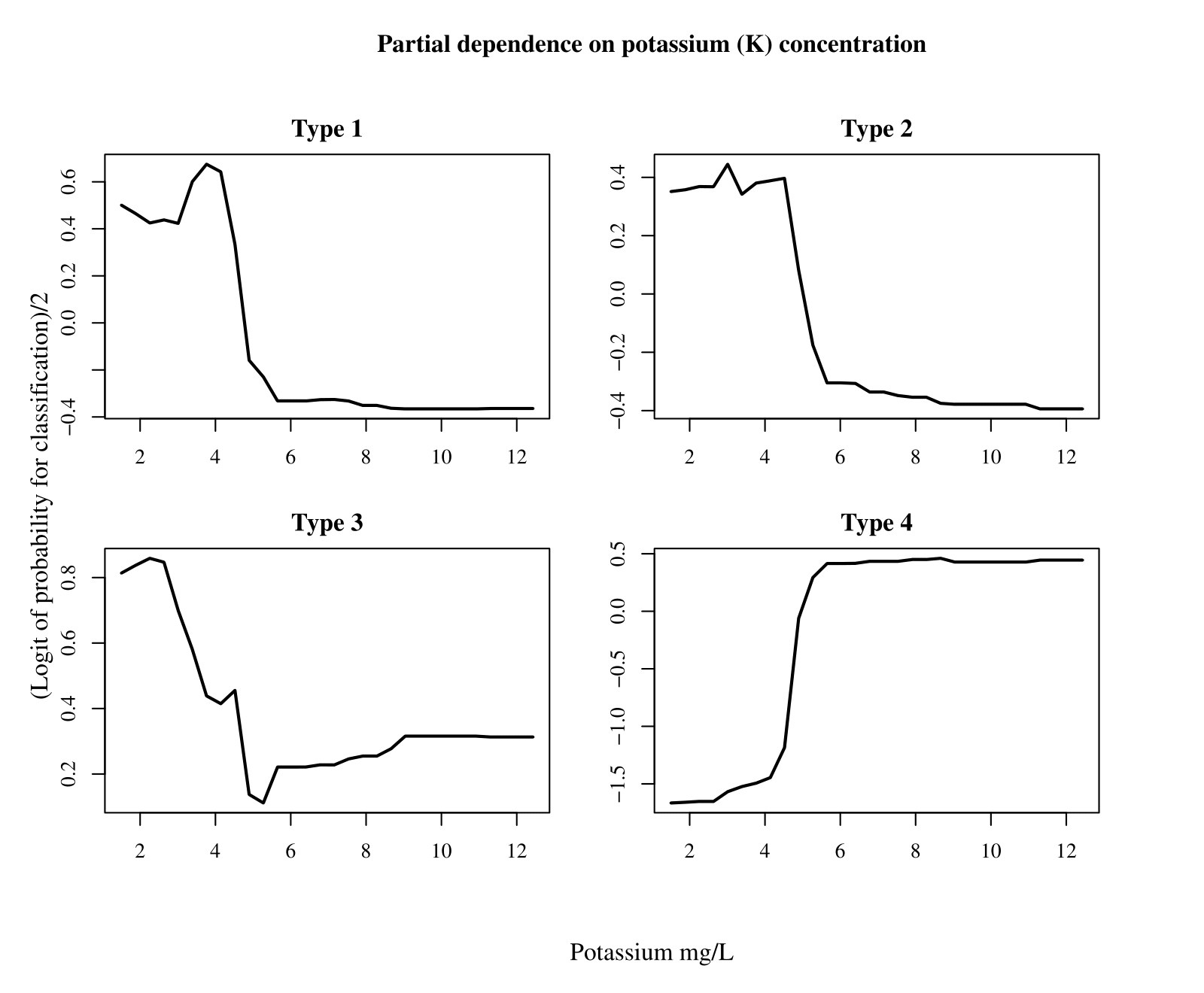
F5 The probability of a pond being classified as Type 4 increases with increasing potassium concentration within the water column.

F6 The probability of a pond being classified as Type 1 increases with increase in floating vegetation


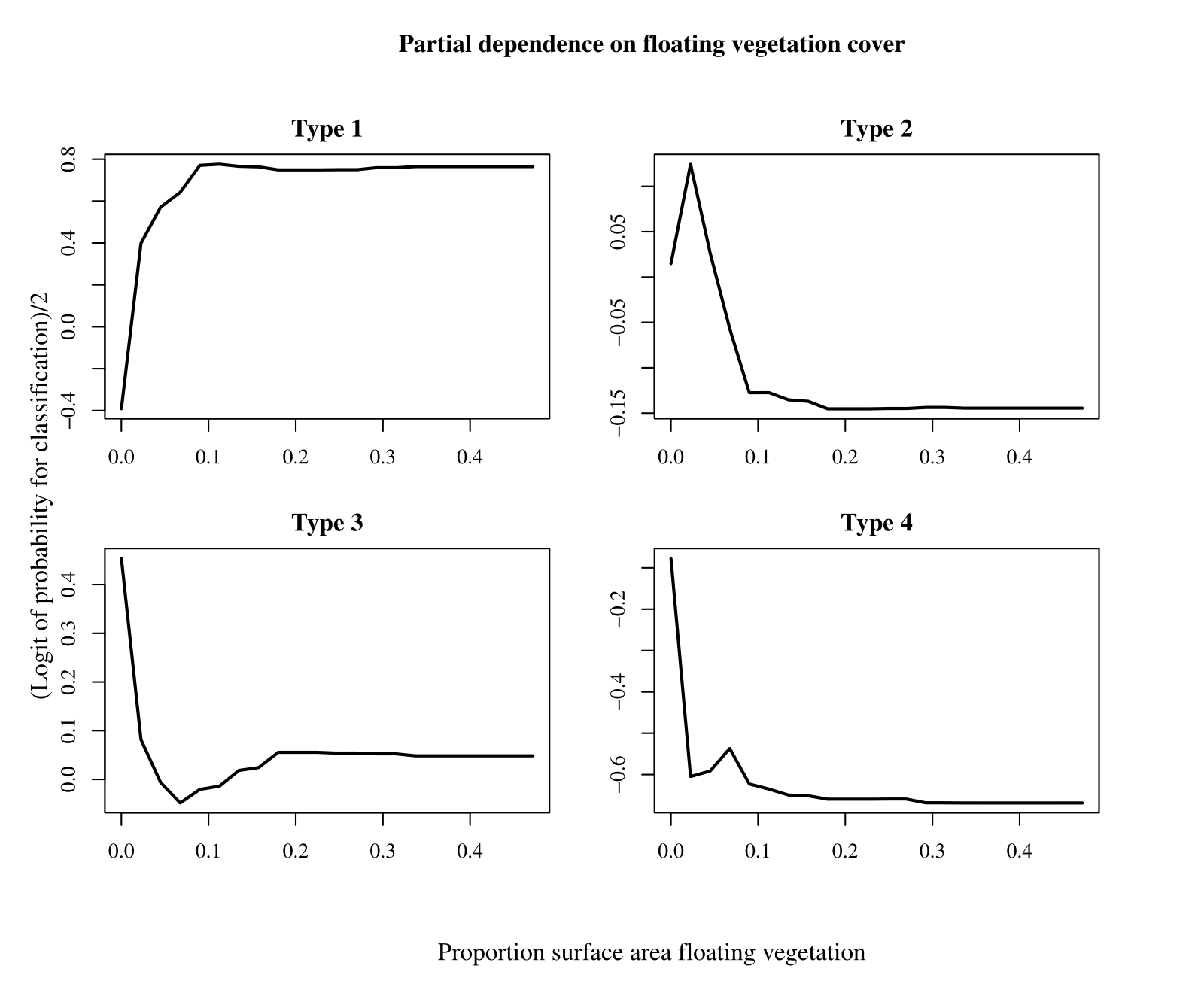


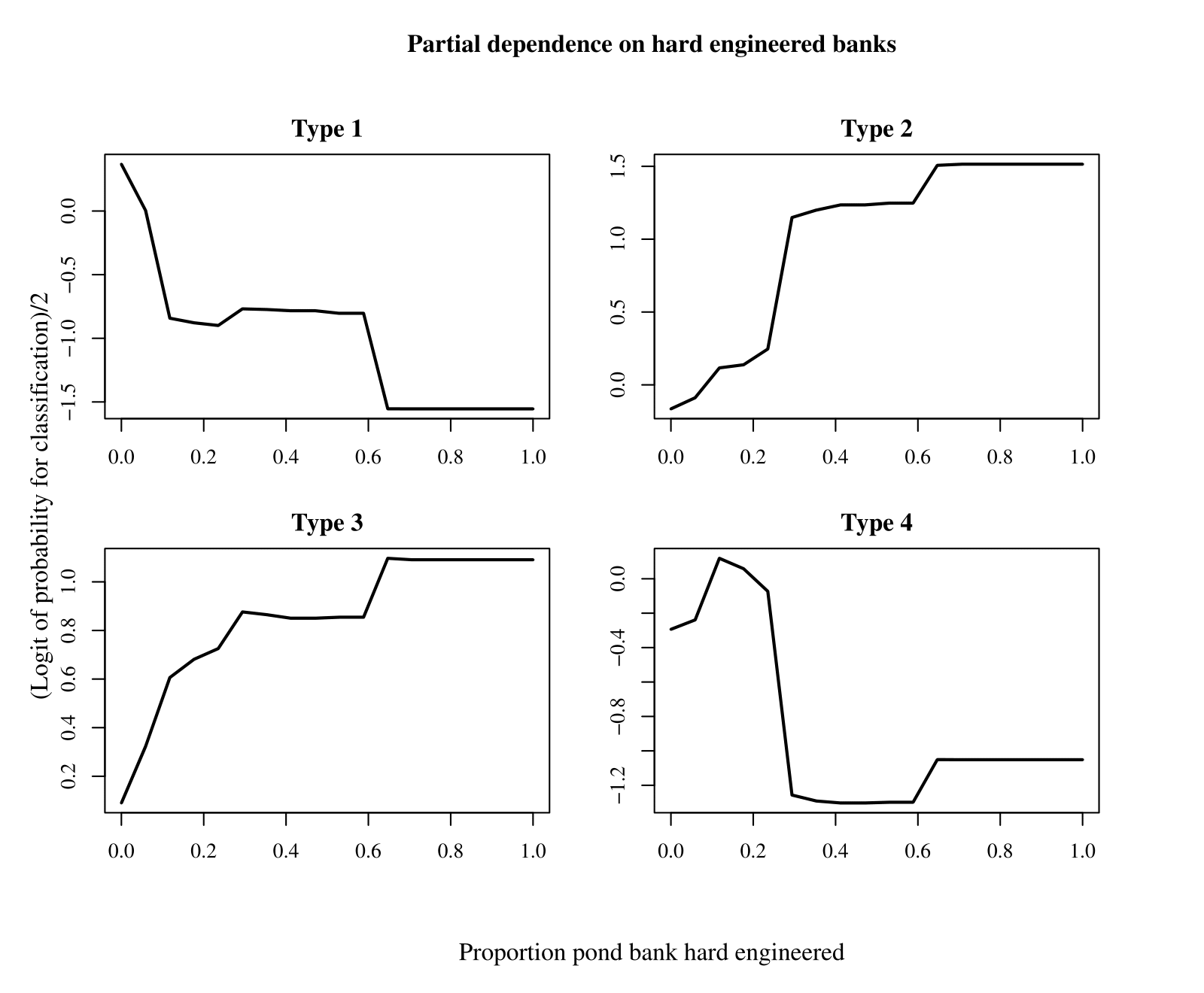
F7 Increases in the proportion of bankside that is hard engineered increases the likelihood that a pond will be classed as Type 2 or 3.


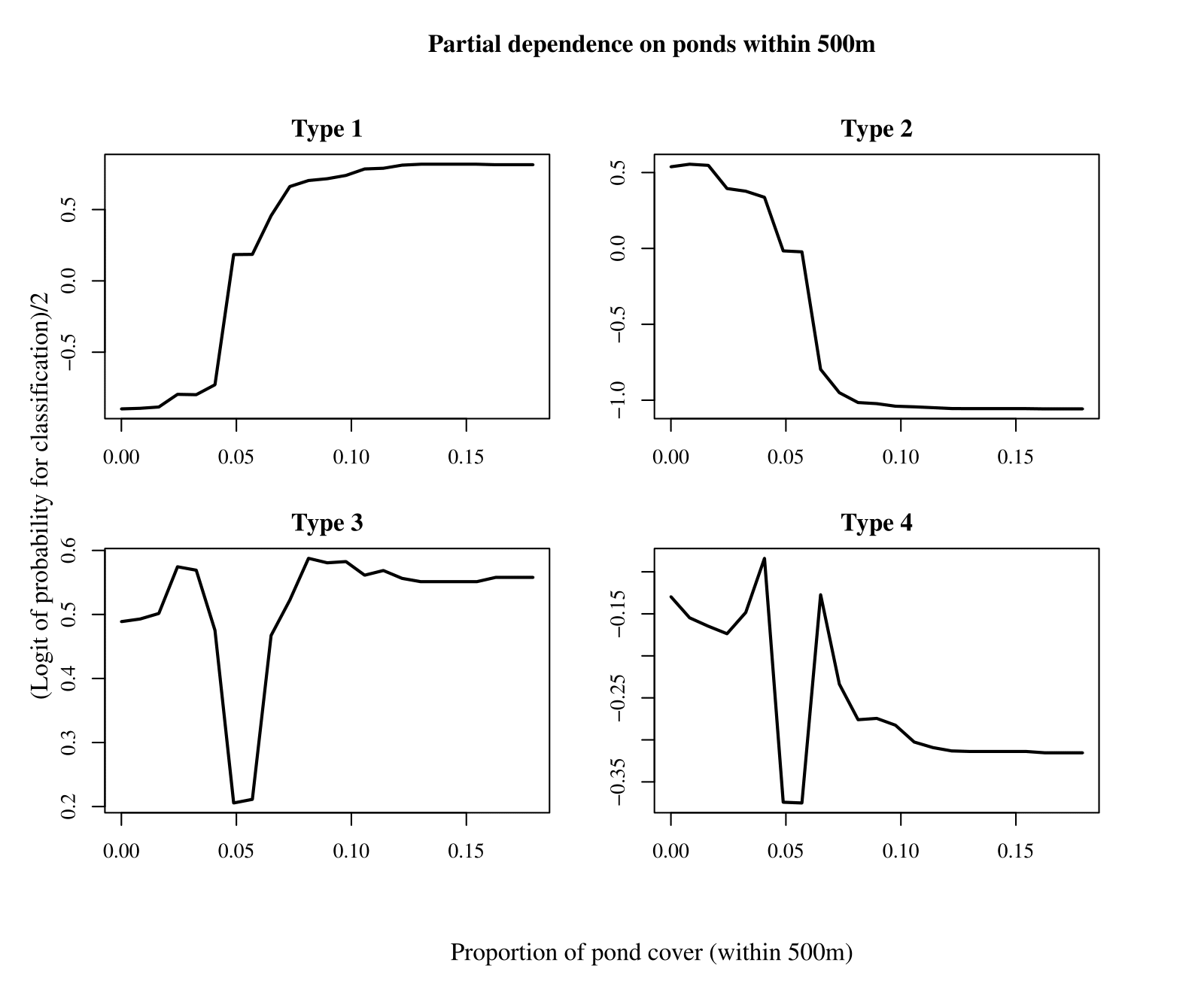
F8 The probability of a site being classified as Type 1 increases when there are more ponds (by surface area) available in the surrounding 500m. The opposite effect is applied to ponds of Type 2.

F9 The probability of pond being classified as Type 1 or Type 3 increases with the amount of scrub (vegetation 0-3m height) within 100m


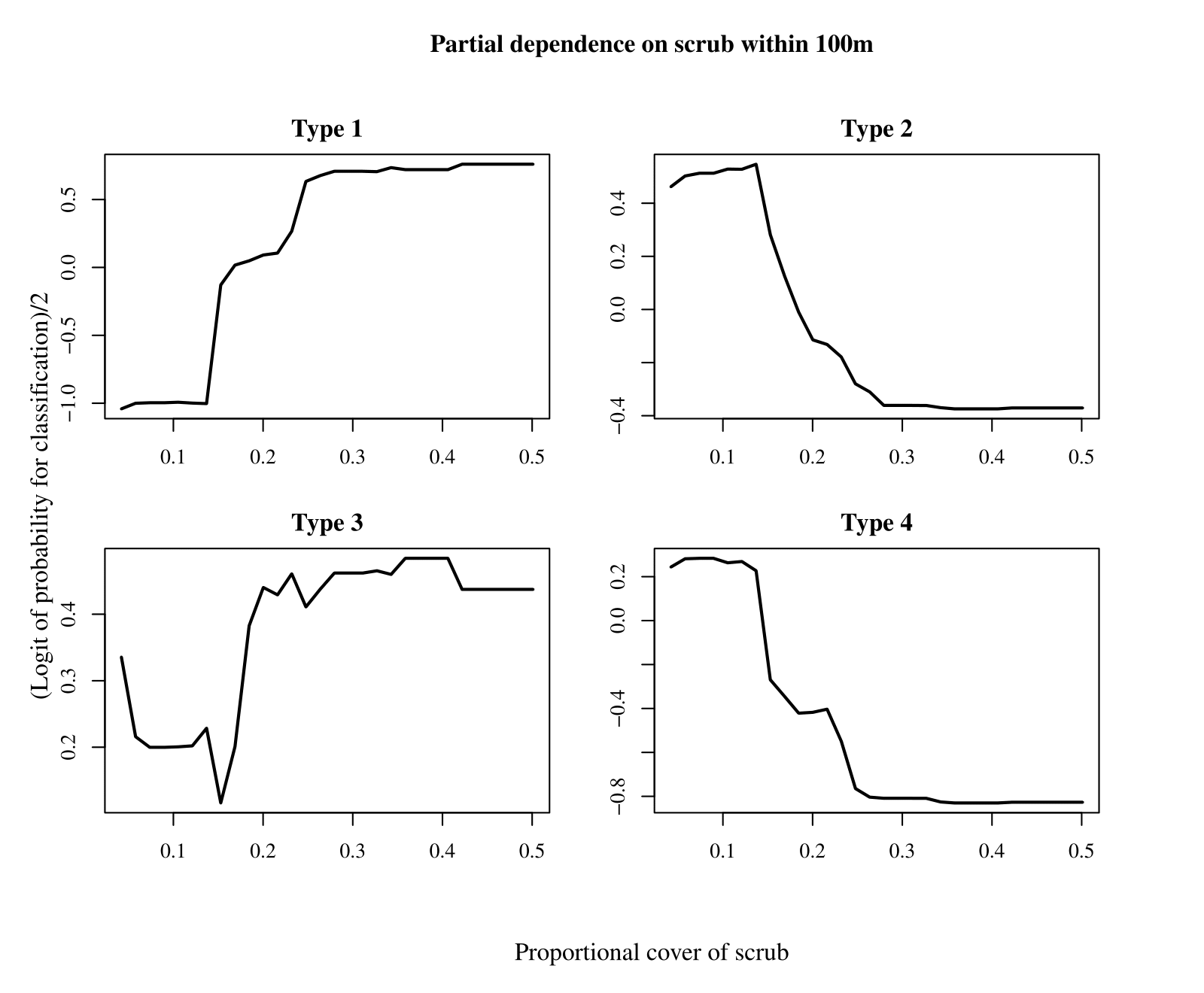


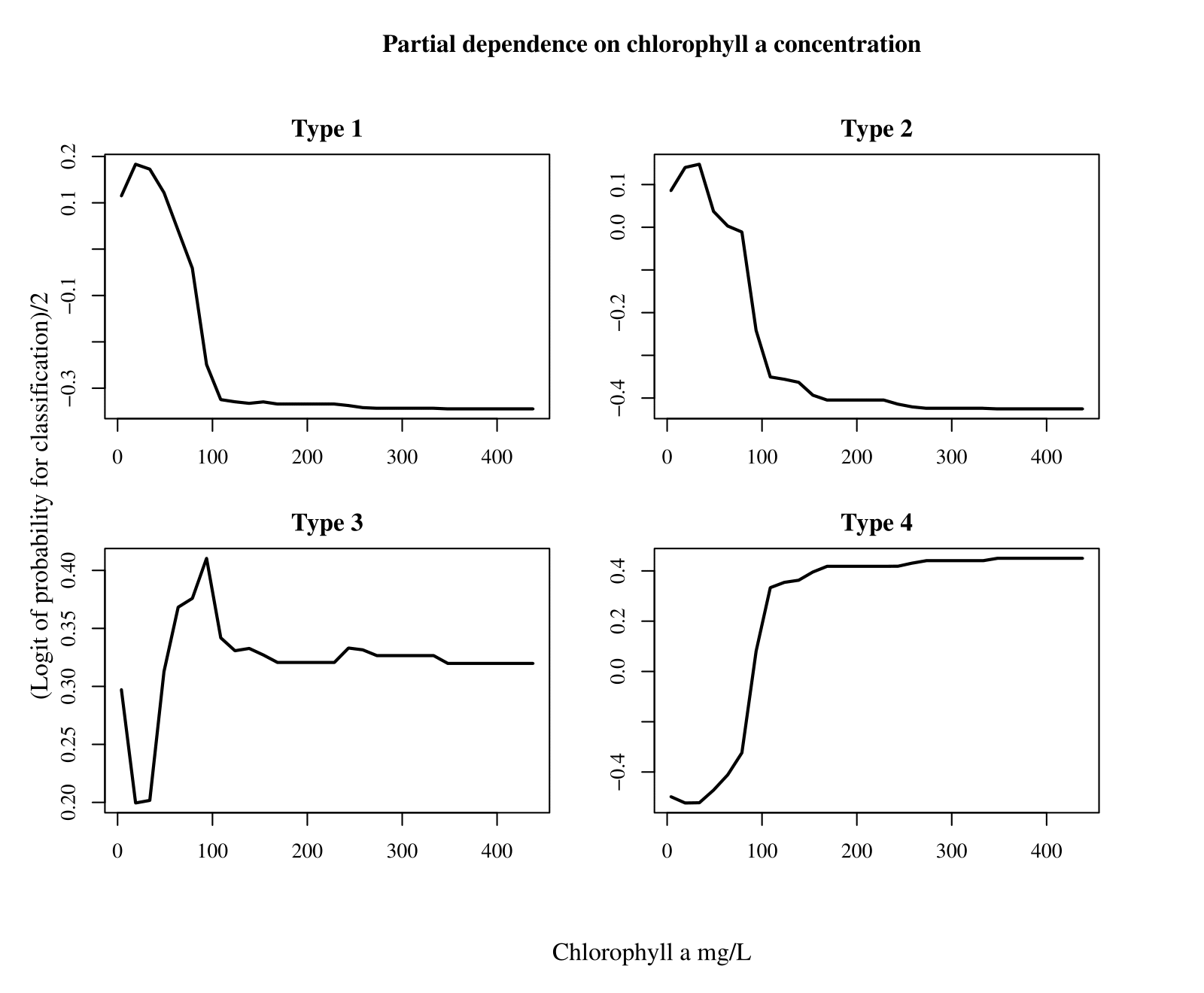
F10 The probability of a site being classified as Type 1 or Type 2 ponds decreases with increases in chlorophyll *a* concentration. The opposite effect is applied to Type 4 ponds.


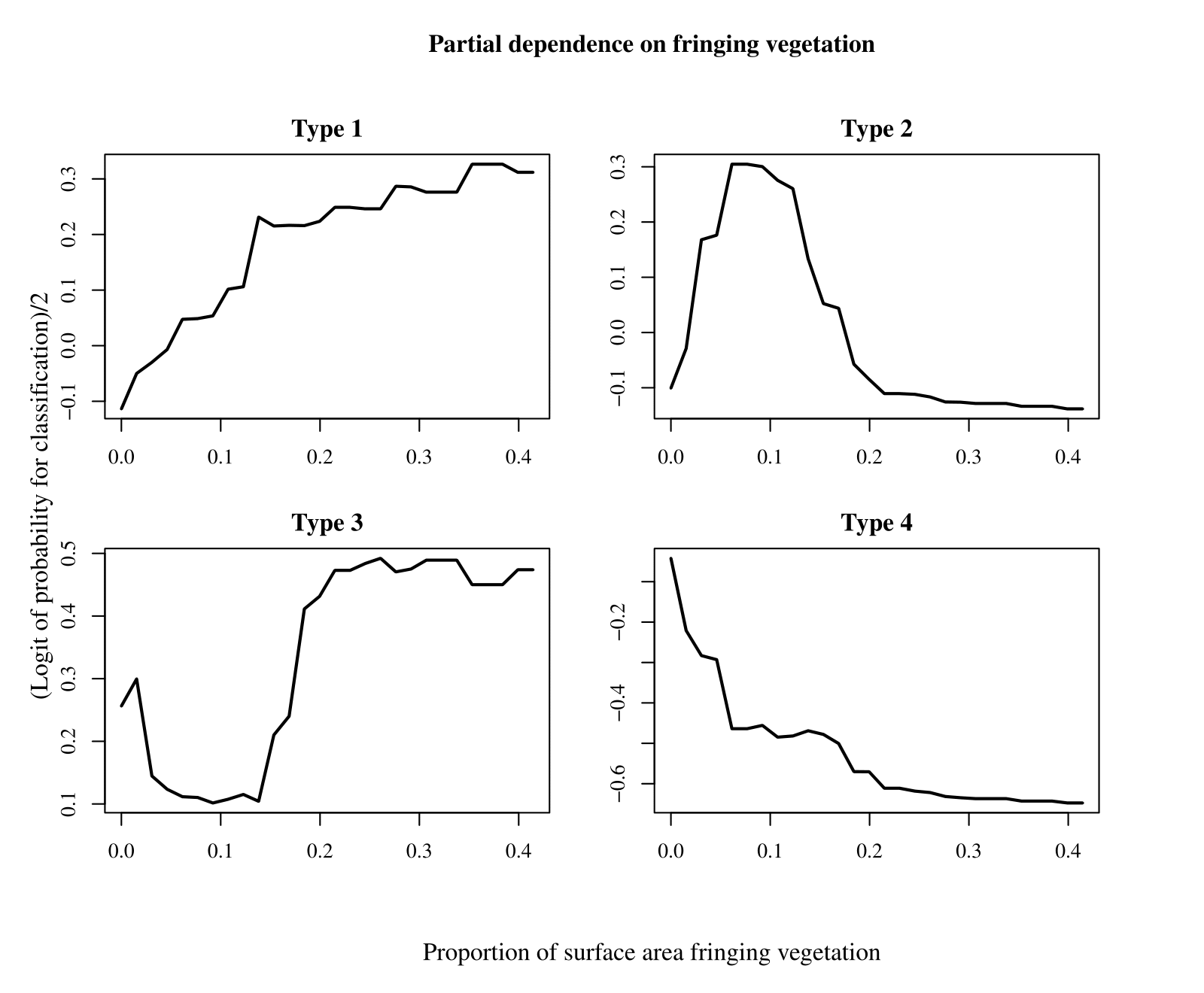
F11 The probability of a site being classified as Type 1 increases with increases in the proportional cover of fringing (marginal) vegetation. The opposite effect is applied to ponds of Type 4.

F12 Highly fluctuating water levels increased the probability of a pond being classified as Type 3.


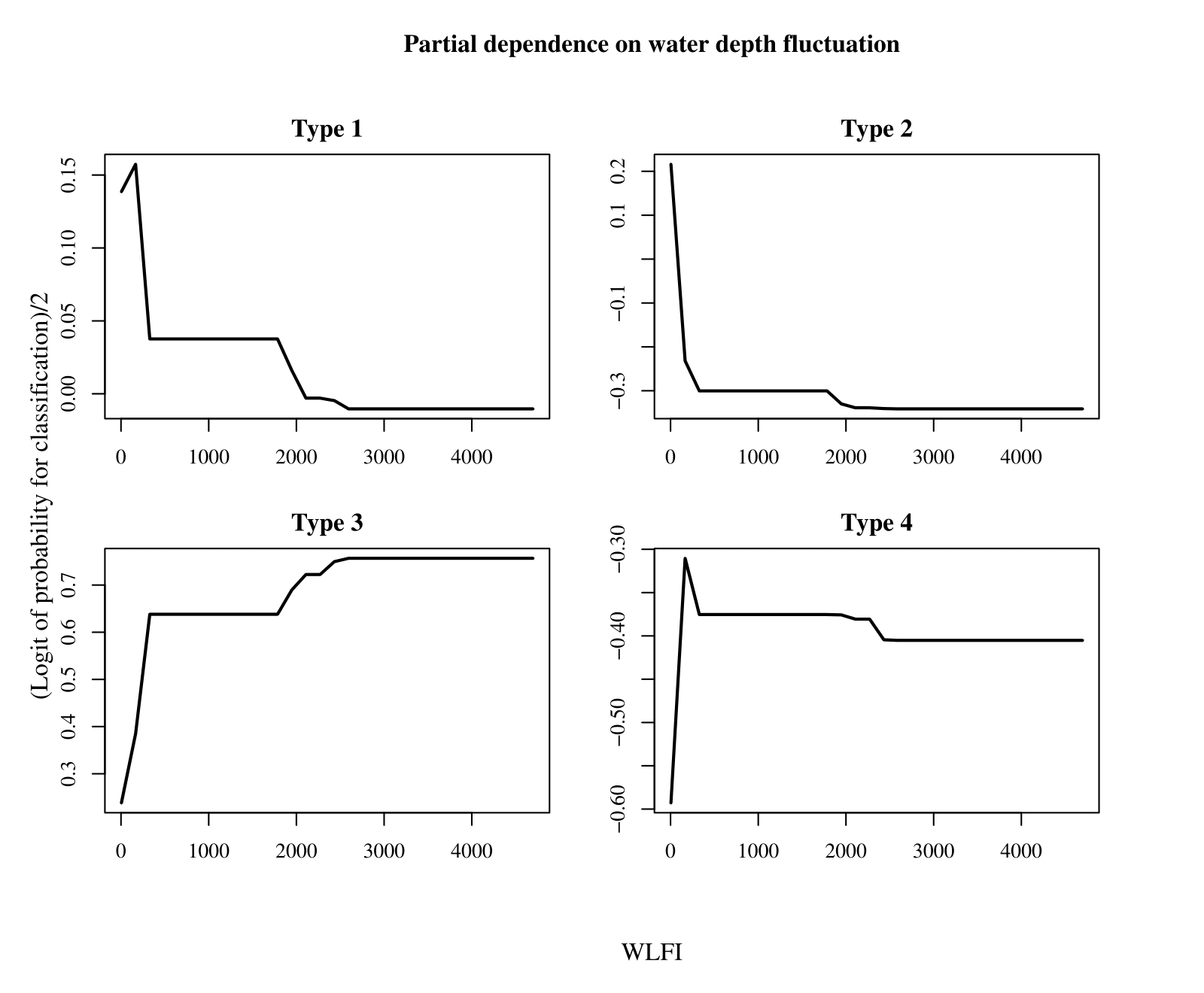


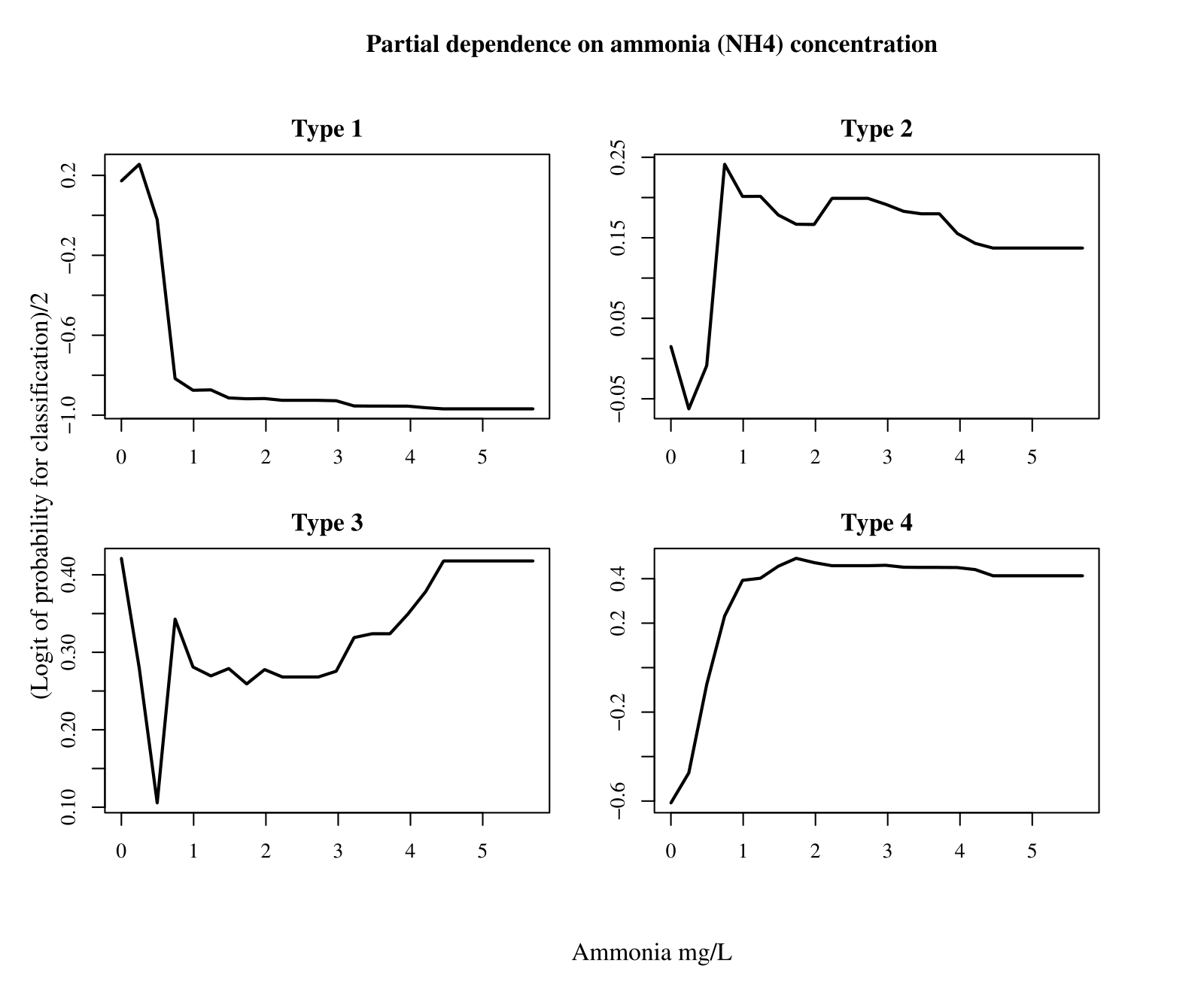
F13 Decreases in the concentration of ammonia decreased the probability of a pond being classified as Type 1, the opposite effect is applied to ponds of Type 4.

F14 Distribution of erroneous class predictions following global model (accuracy 62.7%)


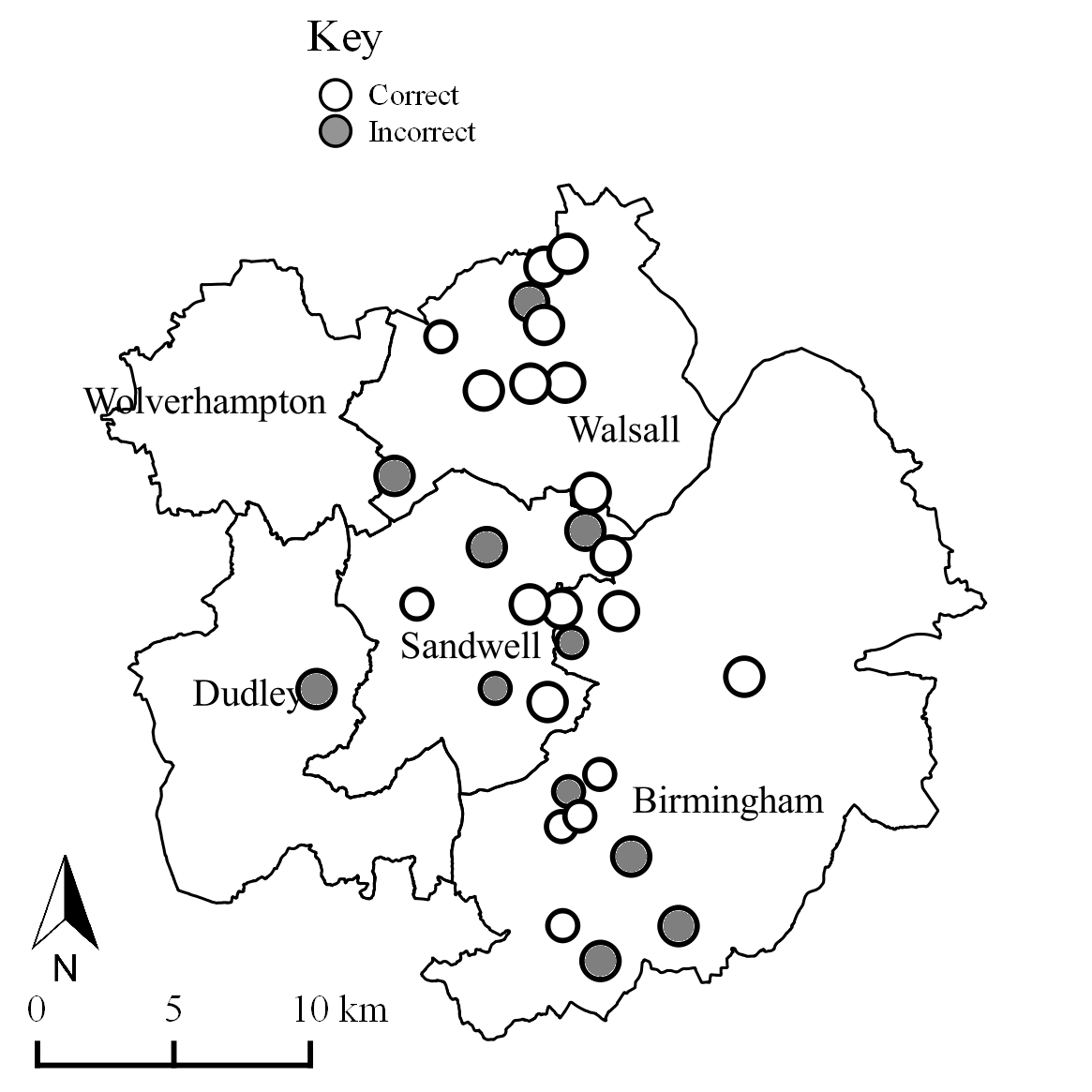


T1 Correlation matrix of local and landscape scale variables (Spearman's Rho)

|  | DC500 | Water | Ponds | IS | IG | Scrub | Tree | Conc. | Shade | Fringe | Float | Mphyte | Spr | Rain | RdBld | Stream | WLFI | Fish | Fished | pH | K | Mg | Ca | Cl | NO_3_ | PO_4_ | SO_4_ | NH_4_ | Alk | Chl-*a* | SS | Fe | Mn |
| --- | --- | --- | --- | --- | --- | --- | --- | --- | --- | --- | --- | --- | --- | --- | --- | --- | --- | --- | --- | --- | --- | --- | --- | --- | --- | --- | --- | --- | --- | --- | --- | --- | --- |
| DC500 |  | 0.52 | 0.67 | -0.57 | 0.07 | 0.28 | 0.42 | -0.22 | -0.10 | 0.08 | 0.12 | 0.26 | 0.21 | -0.19 | 0.28 | -0.27 | 0.00 | -0.12 | -0.10 | -0.06 | 0.01 | -0.05 | 0.10 | -0.06 | -0.10 | -0.20 | 0.14 | -0.01 | 0.17 | -0.11 | -0.02 | -0.11 | 0.05 |
| Water | 0.52 |  | 0.65 | -0.50 | 0.14 | 0.43 | -0.05 | -0.41 | -0.56 | 0.29 | 0.39 | 0.73 | 0.07 | 0.14 | -0.14 | -0.06 | 0.23 | 0.14 | 0.10 | 0.28 | -0.42 | 0.16 | 0.05 | 0.08 | 0.02 | -0.73 | 0.13 | -0.38 | 0.32 | -0.07 | 0.12 | -0.02 | -0.33 |
| Ponds | 0.67 | 0.65 |  | -0.57 | 0.29 | 0.22 | 0.34 | -0.31 | -0.19 | -0.05 | 0.24 | 0.55 | 0.06 | -0.05 | 0.20 | -0.28 | 0.06 | 0.00 | 0.13 | -0.03 | -0.24 | -0.05 | 0.00 | 0.17 | 0.09 | -0.50 | 0.17 | -0.21 | 0.05 | 0.08 | 0.00 | -0.06 | -0.07 |
| IS | -0.57 | -0.50 | -0.57 |  | -0.42 | -0.18 | -0.52 | 0.50 | 0.30 | 0.12 | -0.30 | -0.39 | -0.18 | 0.14 | 0.17 | 0.01 | 0.01 | -0.03 | -0.21 | -0.25 | 0.20 | -0.38 | -0.36 | -0.18 | -0.18 | 0.26 | -0.50 | 0.20 | -0.37 | 0.16 | 0.19 | 0.36 | 0.05 |
| IG | 0.07 | 0.14 | 0.29 | -0.42 |  | -0.29 | 0.09 | -0.18 | -0.02 | -0.03 | 0.29 | 0.04 | -0.06 | 0.27 | -0.09 | 0.00 | -0.07 | -0.09 | 0.04 | -0.15 | 0.13 | 0.31 | 0.28 | -0.11 | 0.10 | -0.08 | 0.30 | -0.09 | 0.24 | 0.13 | -0.17 | -0.06 | 0.20 |
| Scrub | 0.28 | 0.43 | 0.22 | -0.18 | -0.29 |  | -0.14 | -0.31 | -0.48 | 0.53 | 0.39 | 0.51 | 0.36 | -0.21 | -0.06 | -0.04 | 0.04 | 0.22 | 0.36 | 0.26 | 0.05 | 0.32 | -0.06 | 0.25 | -0.08 | -0.47 | 0.38 | -0.26 | 0.06 | -0.21 | -0.15 | -0.16 | -0.38 |
| Tree | 0.42 | -0.05 | 0.34 | -0.52 | 0.09 | -0.14 |  | 0.05 | 0.42 | -0.51 | -0.13 | -0.06 | 0.20 | -0.44 | -0.05 | -0.04 | -0.07 | -0.05 | 0.05 | -0.03 | -0.17 | -0.09 | 0.17 | -0.01 | 0.00 | 0.32 | 0.13 | -0.13 | -0.20 | -0.16 | -0.25 | -0.26 | 0.11 |
| Conc. | -0.22 | -0.41 | -0.31 | 0.50 | -0.18 | -0.31 | 0.05 |  | 0.37 | -0.13 | -0.28 | -0.67 | 0.04 | -0.22 | 0.13 | 0.07 | -0.45 | -0.07 | -0.27 | -0.13 | -0.02 | -0.34 | -0.06 | -0.13 | -0.09 | 0.49 | -0.24 | 0.04 | -0.13 | 0.07 | -0.16 | 0.19 | 0.15 |
| Shade | -0.10 | -0.56 | -0.19 | 0.30 | -0.02 | -0.48 | 0.42 | 0.37 |  | -0.33 | -0.68 | -0.44 | 0.03 | -0.13 | 0.25 | -0.03 | 0.04 | -0.20 | -0.13 | -0.42 | 0.15 | -0.24 | 0.03 | -0.02 | -0.08 | 0.66 | -0.19 | 0.06 | -0.28 | -0.15 | -0.03 | 0.20 | 0.56 |
| Fringe | 0.08 | 0.29 | -0.05 | 0.12 | -0.03 | 0.53 | -0.51 | -0.13 | -0.33 |  | 0.33 | 0.33 | 0.04 | 0.12 | 0.06 | 0.05 | 0.08 | 0.03 | -0.03 | -0.05 | 0.16 | 0.34 | 0.08 | -0.07 | -0.12 | -0.36 | 0.14 | -0.22 | 0.35 | -0.20 | -0.17 | -0.06 | -0.15 |
| Float | 0.12 | 0.39 | 0.24 | -0.30 | 0.29 | 0.39 | -0.13 | -0.28 | -0.68 | 0.33 |  | 0.35 | 0.05 | -0.14 | -0.12 | 0.11 | -0.22 | 0.30 | 0.16 | 0.29 | -0.14 | 0.37 | 0.17 | -0.16 | 0.11 | -0.47 | 0.13 | -0.20 | 0.17 | 0.07 | -0.28 | -0.28 | -0.40 |
| Mphyte | 0.26 | 0.73 | 0.55 | -0.39 | 0.04 | 0.51 | -0.06 | -0.67 | -0.44 | 0.33 | 0.35 |  | 0.18 | 0.10 | -0.16 | -0.21 | 0.49 | 0.07 | 0.16 | 0.20 | -0.31 | 0.24 | -0.06 | 0.18 | 0.16 | -0.76 | 0.19 | -0.33 | 0.08 | -0.08 | 0.03 | -0.09 | -0.39 |
| Spr | 0.21 | 0.07 | 0.06 | -0.18 | -0.06 | 0.36 | 0.20 | 0.04 | 0.03 | 0.04 | 0.05 | 0.18 |  | -0.31 | -0.27 | -0.39 | -0.17 | 0.42 | 0.39 | 0.04 | -0.12 | 0.05 | 0.06 | 0.10 | 0.13 | -0.15 | 0.28 | -0.32 | -0.11 | -0.22 | -0.38 | -0.02 | -0.10 |
| RainRun | -0.19 | 0.14 | -0.05 | 0.14 | 0.27 | -0.21 | -0.44 | -0.22 | -0.13 | 0.12 | -0.14 | 0.10 | -0.31 |  | -0.18 | -0.27 | 0.39 | -0.19 | -0.28 | -0.01 | 0.27 | 0.15 | 0.01 | -0.22 | -0.08 | -0.21 | 0.04 | 0.03 | 0.31 | 0.24 | 0.38 | 0.09 | 0.09 |
| RdBldRun | 0.28 | -0.14 | 0.20 | 0.17 | -0.09 | -0.06 | -0.05 | 0.13 | 0.25 | 0.06 | -0.12 | -0.16 | -0.27 | -0.18 |  | -0.26 | 0.02 | -0.39 | -0.25 | -0.34 | 0.27 | -0.40 | -0.31 | 0.17 | -0.17 | 0.18 | -0.23 | 0.14 | -0.07 | 0.07 | 0.16 | 0.32 | 0.25 |
| Stream | -0.27 | -0.06 | -0.28 | 0.01 | 0.00 | -0.04 | -0.04 | 0.07 | -0.03 | 0.05 | 0.11 | -0.21 | -0.39 | -0.27 | -0.26 |  | -0.21 | 0.16 | 0.12 | -0.04 | -0.06 | 0.25 | 0.23 | 0.04 | -0.03 | 0.16 | -0.02 | 0.17 | 0.10 | -0.13 | -0.06 | -0.08 | 0.05 |
| WLFI | 0.00 | 0.23 | 0.06 | 0.01 | -0.07 | 0.04 | -0.07 | -0.45 | 0.04 | 0.08 | -0.22 | 0.49 | -0.17 | 0.39 | 0.02 | -0.21 |  | -0.42 | -0.33 | -0.04 | 0.09 | 0.00 | -0.16 | 0.02 | -0.32 | -0.20 | -0.18 | 0.01 | 0.08 | -0.04 | 0.34 | 0.03 | -0.07 |
| Fish | -0.12 | 0.14 | 0.00 | -0.03 | -0.09 | 0.22 | -0.05 | -0.07 | -0.20 | 0.03 | 0.30 | 0.07 | 0.42 | -0.19 | -0.39 | 0.16 | -0.42 |  | 0.75 | 0.26 | -0.25 | 0.05 | 0.21 | -0.12 | 0.20 | -0.27 | 0.11 | -0.33 | 0.01 | -0.07 | -0.37 | -0.24 | -0.30 |
| Fished | -0.10 | 0.10 | 0.13 | -0.21 | 0.04 | 0.36 | 0.05 | -0.27 | -0.13 | -0.03 | 0.16 | 0.16 | 0.39 | -0.28 | -0.25 | 0.12 | -0.33 | 0.75 |  | 0.14 | -0.19 | 0.13 | 0.19 | 0.20 | 0.10 | -0.21 | 0.34 | -0.38 | -0.05 | 0.02 | -0.28 | -0.10 | -0.18 |
| pH | -0.06 | 0.28 | -0.03 | -0.25 | -0.15 | 0.26 | -0.03 | -0.13 | -0.42 | -0.05 | 0.29 | 0.20 | 0.04 | -0.01 | -0.34 | -0.04 | -0.04 | 0.26 | 0.14 |  | -0.38 | 0.17 | 0.10 | -0.01 | 0.06 | -0.12 | 0.01 | -0.32 | 0.11 | 0.02 | 0.08 | -0.26 | -0.61 |
| K | 0.01 | -0.42 | -0.24 | 0.20 | 0.13 | 0.05 | -0.17 | -0.02 | 0.15 | 0.16 | -0.14 | -0.31 | -0.12 | 0.27 | 0.27 | -0.06 | 0.09 | -0.25 | -0.19 | -0.38 |  | 0.14 | 0.02 | -0.10 | -0.18 | 0.18 | 0.24 | 0.44 | 0.11 | 0.04 | 0.00 | -0.13 | 0.21 |
| Mg | -0.05 | 0.16 | -0.05 | -0.38 | 0.31 | 0.32 | -0.09 | -0.34 | -0.24 | 0.34 | 0.37 | 0.24 | 0.05 | 0.15 | -0.40 | 0.25 | 0.00 | 0.05 | 0.13 | 0.17 | 0.14 |  | 0.54 | 0.14 | 0.32 | -0.28 | 0.60 | -0.15 | 0.38 | -0.34 | -0.24 | -0.30 | 0.16 |
| Ca | 0.10 | 0.05 | 0.00 | -0.36 | 0.28 | -0.06 | 0.17 | -0.06 | 0.03 | 0.08 | 0.17 | -0.06 | 0.06 | 0.01 | -0.31 | 0.23 | -0.16 | 0.21 | 0.19 | 0.10 | 0.02 | 0.54 |  | -0.18 | 0.08 | -0.04 | 0.44 | -0.02 | 0.58 | -0.21 | -0.20 | -0.39 | 0.16 |
| Cl | -0.06 | 0.08 | 0.17 | -0.18 | -0.11 | 0.25 | -0.01 | -0.13 | -0.02 | -0.07 | -0.16 | 0.18 | 0.10 | -0.22 | 0.17 | 0.04 | 0.02 | -0.12 | 0.20 | -0.01 | -0.10 | 0.14 | -0.18 |  | 0.21 | -0.23 | 0.32 | -0.22 | -0.10 | -0.15 | 0.13 | 0.39 | 0.14 |
| NO_3_ | -0.10 | 0.02 | 0.09 | -0.18 | 0.10 | -0.08 | 0.00 | -0.09 | -0.08 | -0.12 | 0.11 | 0.16 | 0.13 | -0.08 | -0.17 | -0.03 | -0.32 | 0.20 | 0.10 | 0.06 | -0.18 | 0.32 | 0.08 | 0.21 |  | -0.19 | 0.24 | -0.02 | -0.03 | -0.23 | -0.22 | -0.27 | 0.06 |
| PO_4_ | -0.20 | -0.73 | -0.50 | 0.26 | -0.08 | -0.47 | 0.32 | 0.49 | 0.66 | -0.36 | -0.47 | -0.76 | -0.15 | -0.21 | 0.18 | 0.16 | -0.20 | -0.27 | -0.21 | -0.12 | 0.18 | -0.28 | -0.04 | -0.23 | -0.19 |  | -0.34 | 0.16 | -0.19 | 0.01 | -0.04 | -0.04 | 0.28 |
| SO_4_ | 0.14 | 0.13 | 0.17 | -0.50 | 0.30 | 0.38 | 0.13 | -0.24 | -0.19 | 0.14 | 0.13 | 0.19 | 0.28 | 0.04 | -0.23 | -0.02 | -0.18 | 0.11 | 0.34 | 0.01 | 0.24 | 0.60 | 0.44 | 0.32 | 0.24 | -0.34 |  | 0.00 | 0.36 | -0.23 | -0.43 | -0.17 | 0.14 |
| NH_4_ | -0.01 | -0.38 | -0.21 | 0.20 | -0.09 | -0.26 | -0.13 | 0.04 | 0.06 | -0.22 | -0.20 | -0.33 | -0.32 | 0.03 | 0.14 | 0.17 | 0.01 | -0.33 | -0.38 | -0.32 | 0.44 | -0.15 | -0.02 | -0.22 | -0.02 | 0.16 | 0.00 |  | -0.08 | -0.01 | 0.13 | -0.04 | 0.28 |
| Alk | 0.17 | 0.32 | 0.05 | -0.37 | 0.24 | 0.06 | -0.20 | -0.13 | -0.28 | 0.35 | 0.17 | 0.08 | -0.11 | 0.31 | -0.07 | 0.10 | 0.08 | 0.01 | -0.05 | 0.11 | 0.11 | 0.38 | 0.58 | -0.10 | -0.03 | -0.19 | 0.36 | -0.08 |  | 0.11 | -0.12 | -0.25 | 0.03 |
| Chl-*a* | -0.11 | -0.07 | 0.08 | 0.16 | 0.13 | -0.21 | -0.16 | 0.07 | -0.15 | -0.20 | 0.07 | -0.08 | -0.22 | 0.24 | 0.07 | -0.13 | -0.04 | -0.07 | 0.02 | 0.02 | 0.04 | -0.34 | -0.21 | -0.15 | -0.23 | 0.01 | -0.23 | -0.01 | 0.11 |  | 0.24 | 0.24 | -0.19 |
| SS | -0.02 | 0.12 | 0.00 | 0.19 | -0.17 | -0.15 | -0.25 | -0.16 | -0.03 | -0.17 | -0.28 | 0.03 | -0.38 | 0.38 | 0.16 | -0.06 | 0.34 | -0.37 | -0.28 | 0.08 | 0.00 | -0.24 | -0.20 | 0.13 | -0.22 | -0.04 | -0.43 | 0.13 | -0.12 | 0.24 |  | 0.33 | 0.02 |
| Fe | -0.11 | -0.02 | -0.06 | 0.36 | -0.06 | -0.16 | -0.26 | 0.19 | 0.20 | -0.06 | -0.28 | -0.09 | -0.02 | 0.09 | 0.32 | -0.08 | 0.03 | -0.24 | -0.10 | -0.26 | -0.13 | -0.30 | -0.39 | 0.39 | -0.27 | -0.04 | -0.17 | -0.04 | -0.25 | 0.24 | 0.33 |  | 0.35 |
| Mn | 0.05 | -0.33 | -0.07 | 0.05 | 0.20 | -0.38 | 0.11 | 0.15 | 0.56 | -0.15 | -0.40 | -0.39 | -0.10 | 0.09 | 0.25 | 0.05 | -0.07 | -0.30 | -0.18 | -0.61 | 0.21 | 0.16 | 0.16 | 0.14 | 0.06 | 0.28 | 0.14 | 0.28 | 0.03 | -0.19 | 0.02 | 0.35 |  |

DC500 - ponds within (500m); Water - wetland coverage (500m), Ponds - pond coverage (500m); IS - impermeable surfaces (250m); IG - improved grassland (250m); scrub - vegetation <3m height (250m); tree - vegetation >3m height (250m); conc. - proportion of hard engineered bank; Shade - shading from overhanging vegetation; Fringe - fringing vegetation cover (e.g. emergents); Float - floating vegetation cover; Mphyte - macrophyte species richness; Spr - spring fed; Rain - stormwater run-off; RdBld - storm water run-off from roads and buildings; Stream - stream inflow; WLFI - water level fluctuation; Fish - presence of fish; Fished - whether fished

T2 Full macroinvertebrate taxa list with presence (+) pooled across two sampling periods (May-June and August 2009)

| Taxa | 1 | 2 | 3 | 4 | 5 | 6 | 7 | 8 | 9 | 10 | 11 | 12 | 13 | 14 | 15 | 16 | 17 | 18 | 19 | 20 | 21 | 22 | 23 | 24 | 25 | 26 | 27 | 28 | 29 | 30 |
| --- | --- | --- | --- | --- | --- | --- | --- | --- | --- | --- | --- | --- | --- | --- | --- | --- | --- | --- | --- | --- | --- | --- | --- | --- | --- | --- | --- | --- | --- | --- |
| **Amphipoda** |  |  |  |  |  |  |  |  |  |  |  |  |  |  |  |  |  |  |  |  |  |  |  |  |  |  |  |  |  |  |
| *Crangonyx pseudogracilis* | + | + | + |  |  | + | + | + | + | + | + | + | + | + |  | + | + | + | + | + | + | + | + | + | + | + | + | + | + | + |
| *Gammarus lacustris* |  |  |  |  |  |  |  |  |  |  |  |  |  |  |  |  |  |  |  | + |  |  | + |  | + |  |  |  |  | + |
| *Gammarus pulex* |  |  |  |  |  |  |  |  |  |  |  |  |  |  |  |  | + | + |  | + | + |  | + |  | + |  |  |  |  | + |
| **Annelida** |  |  |  |  |  |  |  |  |  |  |  |  |  |  |  |  |  |  |  |  |  |  |  |  |  |  |  |  |  |  |
| Oligochaeta | + | + | + | + | + | + | + | + | + | + | + | + | + | + | + | + | + | + | + | + | + | + | + | + | + | + | + | + | + | + |
| **Bivalvia** |  |  |  |  |  |  |  |  |  |  |  |  |  |  |  |  |  |  |  |  |  |  |  |  |  |  |  |  |  |  |
| Sphaeriidae | + | + | + |  | + | + |  | + | + | + | + | + |  | + |  | + | + | + | + | + | + | + | + | + | + |  | + | + | + | + |
| **Coleoptera** |  |  |  |  |  |  |  |  |  |  |  |  |  |  |  |  |  |  |  |  |  |  |  |  |  |  |  |  |  |  |
| *Acilius sulcatus* |  |  |  |  | + |  |  |  |  |  |  |  | + |  |  |  |  |  | + |  |  | + |  |  |  |  |  |  |  |  |
| *Agabus bipustulatus* |  | + |  |  |  |  |  |  |  | + |  |  |  |  |  |  |  |  |  |  |  | + |  |  |  | + |  | + |  |  |
| *Agabus sturmii* |  |  |  |  |  |  |  |  |  |  |  |  |  |  |  |  |  |  | + | + |  |  | + |  |  |  |  | + |  |  |
| *Anacaena globulus* |  |  |  |  |  |  |  | + |  |  |  |  |  |  |  |  |  |  |  |  |  |  |  |  |  |  |  |  |  |  |
| *Anacaena limbata* |  |  |  |  |  |  |  |  |  | + |  |  |  |  |  |  |  |  |  |  |  | + | + |  |  |  |  | + |  |  |
| *Anacaena lutescens* |  |  |  |  |  |  |  |  |  | + |  |  |  | + |  |  |  |  |  |  | + | + | + |  | + |  |  |  |  |  |
| *Cercyon convexiusculus* | + |  |  |  |  |  |  |  |  |  |  |  |  |  |  |  |  |  |  |  |  |  |  |  |  |  |  |  |  |  |
| *Colymbetes fuscus* |  |  |  |  |  |  |  |  |  |  |  |  | + |  |  |  |  |  | + |  |  |  |  |  |  |  |  | + |  | + |
| *Dytiscus marginalis* |  |  |  |  |  |  |  |  |  |  |  |  |  |  |  |  |  |  |  |  |  |  |  |  | + |  |  |  |  | + |
| *Enochrus testaceus* |  | + |  |  |  |  |  |  |  |  |  | + |  | + |  |  |  | + |  |  | + |  | + |  |  |  | + |  |  |  |
| *Gyrinus substriatus* |  |  |  |  |  |  |  | + |  |  |  |  |  |  |  |  |  |  |  |  |  |  |  |  |  |  |  |  |  |  |
| *Haliplus confinis* |  | + |  |  |  |  |  |  |  |  |  | + |  |  |  |  |  |  |  |  |  |  |  |  |  |  |  |  |  |  |
| *Haliplus immaculatus* |  |  |  |  |  |  |  |  |  |  |  | + |  |  |  |  |  | + |  |  |  |  | + |  |  |  | + |  |  |  |
| *Haliplus lineatocollis* |  | + |  |  |  |  |  |  |  |  |  |  |  |  |  |  |  |  |  |  |  |  |  |  |  |  |  |  | + |  |
| *Haliplus lineolatus* |  |  |  |  |  |  |  |  |  |  |  |  |  |  |  |  |  | + |  |  |  |  | + |  |  |  |  |  |  |  |
| *Haliplus ruficollis* | + | + |  |  |  |  |  |  |  |  |  | + | + | + |  |  |  | + | + |  | + | + | + |  | + |  | + | + | + |  |
| *Helochares lividus* |  |  |  |  |  |  |  |  |  | + |  |  |  |  |  |  |  |  |  |  | + |  |  |  |  |  |  |  |  |  |
| *Helophorus aequalis* |  |  |  |  |  |  |  |  |  | + |  |  |  |  |  |  |  |  |  |  |  | + |  |  | + |  |  |  | + |  |
| *Helophorus brevipalpis* |  |  | + |  |  |  |  |  |  | + | + | + |  |  |  |  | + |  | + | + | + | + | + |  | + |  | + | + |  |  |
| *Helophorus flavipes/obscurus* |  |  |  |  |  |  |  |  |  | + |  | + |  |  |  |  |  |  |  | + | + |  |  |  |  |  |  |  |  |  |
| *Helophorus grandis* |  |  |  |  |  |  |  |  |  |  |  |  |  |  |  |  |  |  | + |  |  |  |  |  |  |  |  | + |  |  |
| *Helophorus minutus* |  |  |  |  |  |  |  |  |  |  |  |  |  |  |  |  |  |  |  |  |  |  | + |  | + | + |  |  | + |  |
| *Helophorus* spp. |  |  |  |  |  | + |  |  |  |  |  |  |  |  |  |  |  |  |  |  |  | + |  |  |  |  |  |  |  |  |
| *Hydraena riparia* |  | + |  |  |  |  |  |  |  |  |  |  |  |  |  |  |  |  |  |  |  |  |  |  | + |  |  |  |  |  |
| *Hydrobius fuscipes* |  |  |  |  |  |  |  |  |  |  |  | + |  |  |  |  |  |  |  | + | + | + |  |  | + |  |  | + |  |  |
| *Hydrochus elongatus* |  |  |  |  |  |  |  |  |  |  |  |  |  |  |  |  |  |  |  |  | + |  |  |  |  |  |  |  |  |  |
| *Hydroglyphus pusillus* |  |  |  |  |  | + | + |  |  |  |  |  |  |  |  |  |  |  |  |  |  |  |  |  |  |  |  |  |  |  |
| *Hydroporus angustatus* |  |  |  |  |  |  |  |  |  |  |  |  |  |  |  |  |  |  | + |  |  |  |  |  |  |  |  | + |  |  |
| *Hydroporus palustris* |  |  |  | + | + |  |  |  |  |  |  |  | + |  |  |  |  |  |  |  |  | + | + |  |  |  |  | + | + | + |
| *Hydroporus planus* |  |  |  |  |  |  |  |  |  |  | + |  |  |  |  |  |  |  |  | + |  |  |  |  |  |  |  | + |  |  |
| *Hydroporus pubescens* |  |  |  |  |  |  |  |  |  |  |  |  |  |  |  |  |  |  |  |  |  |  |  |  |  |  |  | + |  |  |
| *Hygrobia hermanni* |  |  |  |  |  |  |  |  |  |  |  | + |  |  |  |  | + |  |  |  |  |  |  |  |  |  |  |  | + |  |
| *Hygrotus impressopunctatus* |  |  |  |  |  |  |  |  |  |  |  |  |  |  |  |  |  |  |  |  | + |  |  |  |  |  |  |  |  |  |
| *Hygrotus inaequalis* |  |  |  |  |  | + |  |  |  |  | + | + |  |  |  |  |  |  | + |  | + | + |  |  |  |  |  |  | + |  |
| *Hygrotus versicolor* |  | + |  |  |  |  |  |  |  |  |  |  |  |  |  |  |  |  |  |  |  |  |  |  |  |  |  |  |  |  |
| *Hyphydrus ovatus* |  |  |  |  |  |  |  |  |  |  |  | + |  |  |  |  |  |  | + |  |  |  | + |  |  |  |  |  | + |  |
| *Ilybius ater* |  |  |  |  |  |  |  |  |  |  |  | + |  |  |  |  |  |  | + | + |  |  |  |  |  |  |  |  |  |  |
| *Ilybius fuliginosus* |  |  |  |  |  |  |  |  |  |  |  |  |  |  |  |  |  |  |  | + |  |  |  |  |  |  |  | + |  |  |
| *Ilybius quadriguttatus* |  |  |  |  |  |  |  |  |  |  |  | + |  |  |  |  |  |  |  |  | + |  |  |  |  |  |  |  |  |  |
| *Laccobius biguttatus* |  |  |  |  |  |  |  |  |  |  |  | + |  |  |  |  |  |  |  |  |  |  | + |  |  |  |  |  |  |  |
| *Laccobius minutus* |  | + |  |  |  |  |  |  |  |  | + |  |  |  |  |  |  |  |  |  | + | + |  |  |  |  |  |  |  |  |
| *Laccophilus hyalinus* |  |  |  |  |  |  |  |  |  |  |  |  |  |  |  |  |  |  |  |  |  |  | + |  |  |  |  |  |  |  |
| *Laccophilus minutus* |  | + |  |  |  |  |  |  |  |  |  | + |  |  |  |  |  |  |  |  |  | + |  |  |  |  |  |  |  |  |
| Larvae | + | + |  |  | + | + |  | + | + | + | + | + | + | + |  |  | + | + | + | + | + | + | + | + | + | + | + | + | + | + |
| *Noterus clavicornis* |  | + | + |  |  | + |  |  |  | + | + | + | + | + |  |  |  | + |  |  | + | + | + |  | + |  | + |  | + |  |
| *Ochthebius minimus* |  |  |  |  |  |  |  |  |  |  |  |  |  |  |  |  |  |  |  |  |  |  |  |  |  |  |  |  | + |  |
| *Rhantus exsoletus* |  |  |  |  |  |  |  |  |  |  |  |  |  |  |  |  |  |  |  |  |  | + |  |  |  |  |  |  |  |  |
| *Rhantus suturalis* |  |  |  |  |  |  |  |  |  |  |  |  |  |  |  |  |  |  |  |  |  |  |  |  |  |  |  | + |  |  |
| *Suphrodytes dorsalis* |  |  |  |  |  |  |  |  |  |  |  | + |  |  |  |  |  |  |  |  |  |  |  |  |  |  |  |  |  |  |
| **Diptera** |  |  |  |  |  |  |  |  |  |  |  |  |  |  |  |  |  |  |  |  |  |  |  |  |  |  |  |  |  |  |
| Ceratopogonidae | + | + |  |  |  | + |  |  | + | + | + | + |  | + | + |  | + | + | + |  | + | + | + | + | + |  | + |  | + | + |
| Chaoboridae |  |  | + |  | + |  | + |  |  |  | + | + | + | + |  |  |  | + | + | + |  | + | + |  |  |  |  | + | + | + |
| Chironomidae | + | + | + | + | + | + | + | + | + | + | + | + | + | + | + | + | + | + | + | + | + | + | + | + | + | + | + | + | + | + |
| Culicidae |  |  |  | + |  |  |  |  |  | + | + |  | + | + |  |  |  |  | + | + | + | + | + |  | + | + | + | + | + | + |
| Dixidae |  | + |  | + |  | + |  | + |  | + |  | + |  | + | + |  |  | + | + | + | + | + | + | + | + | + | + |  | + | + |
| Empididae |  |  |  |  |  |  |  |  |  |  |  |  |  |  |  |  |  |  |  |  |  |  |  |  | + |  |  |  |  |  |
| Ephydridae | + |  |  |  |  |  |  |  | + |  |  |  |  |  |  |  |  |  |  |  |  |  |  |  |  |  |  | + |  |  |
| Limonidae/Pedicidae |  |  |  |  |  |  | + |  | + | + | + |  | + |  |  |  |  |  | + | + |  |  |  |  | + | + |  |  |  |  |
| Psychodidae | + |  |  |  |  | + |  |  |  |  |  |  | + |  | + |  |  | + | + | + | + | + | + |  | + |  | + | + | + | + |
| Ptychopteridae | + | + |  |  |  |  |  |  |  |  |  |  |  |  |  |  |  |  |  | + |  |  |  |  |  |  |  |  | + |  |
| Sciomyzidae |  | + |  |  |  |  |  |  |  |  |  |  |  |  |  |  |  |  |  |  |  |  |  |  | + |  |  |  |  |  |
| Stratiomyidae |  |  |  |  |  |  |  |  |  |  |  |  | + | + |  |  |  |  | + |  |  |  | + |  |  |  | + |  |  |  |
| Syrphidae |  |  | + | + |  | + |  |  |  |  |  |  | + |  |  |  |  |  | + |  | + | + |  |  | + |  |  | + |  | + |
| Tabanidae |  | + |  |  |  |  |  |  |  | + |  |  |  |  |  |  |  |  |  |  | + | + |  |  |  |  |  |  |  |  |
| Tipulidae | + | + |  |  |  | + | + |  | + | + | + | + | + | + |  | + |  | + |  | + | + | + | + |  | + | + | + | + | + |  |
| **Ephemeroptera** |  |  |  |  |  |  |  |  |  |  |  |  |  |  |  |  |  |  |  |  |  |  |  |  |  |  |  |  |  |  |
| Baetidae spp. |  |  |  |  |  |  |  |  |  |  |  |  |  |  |  |  |  |  |  |  |  |  |  |  |  |  |  |  |  | + |
| *Caenis horaria* | + | + |  |  |  |  |  |  | + |  |  | + |  |  |  |  | + | + |  |  |  |  | + | + | + |  |  |  |  |  |
| *Caenis luctuosa* |  |  |  |  |  |  |  |  |  |  |  |  |  |  |  |  |  |  |  |  |  |  |  |  | + |  |  |  |  |  |
| *Caenis robusta* |  | + |  |  |  |  |  |  |  |  |  |  |  |  |  |  | + | + |  |  | + |  |  |  |  |  | + |  |  |  |
| *Cloeon dipterum* | + | + | + | + | + | + |  | + | + | + | + | + | + | + | + |  | + | + | + | + | + | + | + | + | + | + | + |  | + |  |
| **Gastropoda** |  |  |  |  |  |  |  |  |  |  |  |  |  |  |  |  |  |  |  |  |  |  |  |  |  |  |  |  |  |  |
| *Acroloxus lacustris* | + | + |  |  |  | + |  | + |  |  |  |  |  |  | + | + |  | + | + |  |  | + | + | + | + |  | + |  |  | + |
| *Anisus vortex* |  |  |  |  |  |  |  |  | + |  |  |  |  |  | + |  |  | + |  |  | + |  | + | + |  |  |  | + |  |  |
| *Armiger crista* | + | + |  | + | + | + |  |  |  |  |  | + |  |  |  | + |  | + | + | + | + |  | + | + | + |  |  |  | + | + |
| *Bithynia leachii* |  |  |  |  |  |  |  |  | + |  |  |  |  |  |  |  |  |  |  |  |  |  |  |  | + |  |  |  |  |  |
| *Bithynia* spp. |  |  |  |  |  |  |  |  |  |  |  |  |  |  |  |  |  |  |  |  |  |  |  |  |  |  | + |  |  |  |
| *Bithynia tentaculata* |  | + |  |  |  |  |  |  | + |  |  |  |  | + | + |  | + |  |  | + | + |  |  | + | + | + | + |  |  |  |
| *Gyraulus albus* | + | + |  |  |  |  |  | + | + | + | + | + |  | + |  |  | + | + | + |  | + |  | + | + | + |  | + | + | + |  |
| *Hippeutis complanatus* |  | + |  |  |  | + |  | + | + |  | + |  | + | + | + | + | + | + | + |  |  |  | + | + |  | + | + |  |  |  |
| *Lymnaea palustris* |  |  |  |  |  |  |  |  | + |  |  |  |  |  |  |  |  |  |  |  |  |  |  |  |  |  |  |  |  |  |
| *Lymnaea stagnalis* | + | + |  |  |  |  |  |  | + | + |  | + |  | + |  |  | + | + |  |  |  |  | + |  | + |  | + | + | + |  |
| *Physa fontinalis* | + | + |  | + | + |  |  |  |  |  | + |  |  |  |  |  | + | + | + | + | + |  | + |  |  | + |  |  |  | + |
| *Planorbarius corneus* | + |  |  |  |  |  |  |  |  |  | + | + |  | + |  | + | + |  |  | + |  | + | + |  | + | + | + | + | + |  |
| *Planorbis carinatus* |  |  |  |  |  |  |  |  |  |  |  |  |  | + |  |  |  | + |  |  |  | + |  |  |  |  | + | + |  |  |
| *Planorbis planorbis* | + | + |  |  |  |  |  |  |  |  |  | + |  | + |  |  |  | + |  |  | + | + | + |  | + |  | + | + |  |  |
| *Potamopyrgus antipodarum* | + | + |  |  |  |  |  | + |  |  |  |  |  |  |  | + |  | + |  | + | + |  | + | + | + |  | + |  |  | + |
| *Radix auricularia* | + |  |  |  |  |  |  |  |  |  |  |  |  |  |  |  |  |  |  |  |  |  |  |  |  |  | + |  |  |  |
| *Radix balthica* | + | + |  |  |  |  |  | + | + |  | + | + |  | + | + |  | + | + |  |  | + | + | + |  |  | + | + | + | + |  |
| *Valvata piscinalis* |  | + |  | + |  |  |  |  | + |  |  |  |  |  |  |  | + |  |  |  |  |  | + |  |  |  |  |  |  |  |
| **Hemiptera** |  |  |  |  |  |  |  |  |  |  |  |  |  |  |  |  |  |  |  |  |  |  |  |  |  |  |  |  |  |  |
| *Callicorixa praeusta* |  |  |  |  |  |  | + |  | + | + | + | + | + |  |  |  |  |  |  |  |  | + |  |  |  |  |  |  | + |  |
| *Callicorixa wollastoni* |  |  |  |  |  |  |  |  |  |  |  |  |  |  |  |  |  |  | + |  |  |  |  |  |  |  |  |  |  |  |
| *Corixa dentipes* |  |  |  |  |  |  | + |  |  |  | + | + |  |  |  |  |  |  |  |  |  |  |  |  |  |  |  |  |  |  |
| *Corixa panzeri* |  |  |  |  |  |  |  |  | + | + |  | + | + |  |  |  | + |  |  |  |  |  |  |  |  |  |  |  |  |  |
| *Corixa punctata* |  |  |  |  |  |  |  |  |  |  | + | + | + |  |  |  |  |  | + |  |  |  |  |  |  |  |  |  |  |  |
| Corixidae (nymphs) | + | + |  | + | + | + | + | + | + | + | + | + | + | + | + | + | + | + | + | + | + | + | + | + | + |  |  | + | + | + |
| *Cymatia bonsdorffi* |  |  |  |  |  | + |  |  |  |  |  | + |  |  |  |  | + |  |  |  |  |  |  |  |  |  |  |  |  |  |
| *Cymatia coleoptrata* |  |  |  |  |  |  |  |  |  |  |  | + |  |  |  |  |  |  |  |  |  |  | + |  |  | + |  |  |  |  |
| *Gerridae nymph* | + | + |  |  |  | + |  | + | + | + |  | + |  | + |  |  |  |  | + | + | + | + | + |  | + |  | + |  | + | + |
| *Gerris lacustris* |  |  |  |  |  | + |  | + | + | + | + |  |  | + |  |  |  |  |  | + |  |  | + |  | + |  | + |  |  |  |
| *Gerris odontogaster* |  |  |  |  |  |  |  |  |  |  |  | + |  | + |  |  |  |  |  |  |  |  | + |  |  |  |  |  |  |  |
| *Hesperocorixa linnei* |  |  |  | + |  |  |  |  |  |  |  | + |  | + |  |  |  |  | + |  | + |  | + |  |  |  |  |  |  |  |
| *Hesperocorixa sahlbergi* |  |  | + |  | + |  |  |  |  |  |  |  | + |  |  |  |  |  | + | + |  | + |  | + |  |  |  |  |  | + |
| *Hydrometra stagnorum* |  |  |  |  |  |  |  | + |  | + | + |  | + |  |  |  |  | + | + | + |  | + | + |  | + |  | + | + |  |  |
| Hydrometridae (nymphs) |  | + |  |  |  |  |  | + | + |  | + |  | + |  |  |  | + | + | + | + |  | + | + | + | + |  | + | + |  |  |
| *Ilycoris* spp. (nymphs) | + |  |  |  |  |  |  |  |  |  |  | + |  | + |  |  |  |  |  |  | + |  | + |  |  |  | + |  |  |  |
| *Ilyocoris cimicoides* |  |  |  |  |  |  |  |  |  |  |  | + |  | + |  |  |  |  |  |  | + |  |  |  |  |  | + |  |  |  |
| *Mesovelia furcata* |  |  |  |  |  |  |  |  |  |  |  |  |  |  |  |  |  |  |  |  |  |  |  |  | + |  |  |  |  |  |
| *Micronecta poweri* |  |  |  |  |  |  |  |  |  | + |  |  |  |  |  |  |  | + |  |  |  |  | + |  |  |  |  |  |  |  |
| *Micronecta scholtzi* | + |  |  |  |  |  | + |  | + | + |  |  |  |  |  |  |  |  |  |  | + |  | + | + | + |  |  |  |  |  |
| *Microvelia reticulata* |  | + |  |  |  |  |  |  |  |  |  | + |  |  |  |  |  |  |  |  |  |  |  |  | + |  | + |  |  |  |
| *Nepa cinerea* |  |  |  |  |  |  |  | + |  |  |  |  |  |  |  |  |  |  |  | + |  |  |  |  | + |  | + |  |  |  |
| Nepidae spp. (nymphs) |  | + |  |  |  |  |  |  | + | + |  |  |  | + |  |  |  |  |  | + |  |  |  |  |  |  | + | + |  |  |
| *Notonecta glauca* |  | + | + |  |  | + |  | + |  | + | + | + | + | + |  |  | + | + | + |  |  | + | + |  | + |  | + |  | + |  |
| *Notonecta maculata* |  |  |  |  |  |  |  |  |  |  | + |  |  |  |  |  |  |  |  |  |  |  |  |  |  |  |  |  |  |  |
| Notonectidae (nymph) |  | + |  |  | + | + | + | + | + | + | + | + | + | + |  |  |  | + | + |  | + | + | + |  | + |  | + |  | + | + |
| *Plea leachi* |  |  |  |  |  |  |  |  |  |  |  |  |  |  |  |  |  |  |  |  |  |  |  |  |  |  | + |  |  |  |
| Pleidae spp. (nymphs) |  | + |  |  |  |  |  |  |  |  |  |  |  |  |  |  |  |  |  |  | + |  |  |  |  |  |  |  |  |  |
| *Ranatra linearis* | + |  |  |  |  |  |  |  |  |  |  | + |  | + |  |  |  |  |  |  | + |  |  |  | + |  | + |  |  |  |
| *Sigara concinna* |  |  |  |  |  |  | + |  |  |  |  | + |  |  |  |  |  |  |  |  |  |  |  |  |  |  |  |  |  |  |
| *Sigara distincta* |  |  | + |  |  | + | + |  | + | + | + | + | + |  |  |  | + | + |  |  | + |  |  |  |  |  |  |  | + |  |
| *Sigara dorsalis* | + | + | + |  |  | + | + |  | + | + | + | + | + | + | + |  | + | + |  |  | + | + | + |  |  |  |  |  | + |  |
| *Sigara falleni* |  |  | + |  |  | + | + |  | + | + | + | + | + | + |  |  |  |  |  |  | + |  | + | + |  |  |  |  |  |  |
| *Sigara fossarum* |  |  |  |  |  |  |  |  |  |  | + | + |  |  |  |  |  |  |  |  |  |  | + |  |  |  |  |  |  |  |
| *Sigara lateralis* |  |  | + |  |  | + | + |  | + |  | + |  | + |  |  | + |  |  |  |  | + |  |  |  |  | + |  |  |  |  |
| *Sigara limitata* |  | + |  |  |  |  |  |  |  |  | + |  |  |  |  |  |  |  |  |  | + |  |  |  |  | + |  |  | + |  |
| *Sigara nigrolineata* |  |  | + |  |  |  |  |  |  |  |  |  |  |  |  |  |  |  |  |  |  |  |  |  |  |  |  |  |  | + |
| *Velia caprai* |  |  |  |  |  |  |  |  |  |  |  |  |  |  |  |  |  |  | + | + |  |  |  |  |  |  |  |  |  |  |
| Veliidae spp. (nymphs) |  |  |  |  | + |  |  | + |  |  |  | + |  |  |  |  |  |  | + | + |  |  | + |  | + |  |  | + |  | + |
| **Hirudinea** |  |  |  |  |  |  |  |  |  |  |  |  |  |  |  |  |  |  |  |  |  |  |  |  |  |  |  |  |  |  |
| *Erpobdella octoculata* |  | + |  |  |  | + | + | + | + |  |  |  | + |  |  |  |  | + | + | + | + |  | + | + | + | + |  |  |  |  |
| *Erpobdella* spp. |  |  |  |  |  | + |  |  |  |  |  |  |  |  |  |  |  |  |  |  |  | + |  |  |  |  |  |  |  |  |
| *Erpobdella testacea* |  | + |  |  |  | + |  |  | + |  |  |  | + | + |  |  |  |  | + | + | + |  |  |  |  | + | + |  |  |  |
| *Glossiphonia complanata* |  |  |  |  |  |  |  |  |  |  |  |  | + |  |  |  | + | + |  | + |  |  | + |  |  | + | + |  |  |  |
| *Glossiphonia heteroclita* |  | + |  |  |  | + |  |  |  |  |  | + |  | + |  | + | + |  |  |  | + |  | + |  |  | + | + |  | + |  |
| *Glossiphonidae spp.* |  |  | + |  |  |  |  |  |  |  |  |  |  |  |  |  |  |  |  |  |  |  |  |  |  |  |  |  |  |  |
| *Helobdella stagnalis* |  | + | + | + | + | + | + | + | + |  | + | + | + |  | + | + | + | + | + | + | + | + | + | + | + | + | + |  | + | + |
| *Hemiclepsis marginata* |  |  |  |  |  | + |  | + |  |  | + |  |  |  |  | + |  | + |  |  |  |  | + |  | + | + | + |  |  |  |
| *Piscicola geometra* |  |  |  |  |  |  |  |  |  |  |  |  |  |  |  |  |  | + |  |  | + |  | + |  | + |  | + |  |  |  |
| *Theromyzon tessulatum* | + |  | + |  |  | + |  |  | + |  | + | + | + | + | + |  | + | + | + | + | + | + | + | + |  | + | + |  | + |  |
| **Isopoda** |  |  |  |  |  |  |  |  |  |  |  |  |  |  |  |  |  |  |  |  |  |  |  |  |  |  |  |  |  |  |
| *Asellus aquaticus* | + | + |  | + | + | + | + | + | + |  | + | + | + | + | + | + | + | + | + | + | + | + | + | + | + | + | + | + | + | + |
| **Megaloptera** |  |  |  |  |  |  |  |  |  |  |  |  |  |  |  |  |  |  |  |  |  |  |  |  |  |  |  |  |  |  |
| *Sialis lutaria* | + | + |  |  |  |  |  | + | + | + | + |  |  |  | + |  |  | + |  | + | + | + | + | + | + |  | + |  |  |  |
| **Odonata** |  |  |  |  |  |  |  |  |  |  |  |  |  |  |  |  |  |  |  |  |  |  |  |  |  |  |  |  |  |  |
| *Aeshna cyanea* |  |  |  |  |  |  |  |  |  | + |  |  |  |  |  |  |  |  | + |  |  | + |  |  |  |  |  | + |  |  |
| *Aeshna grandis* |  |  |  |  |  |  |  | + |  | + |  | + |  | + |  |  |  | + |  | + |  | + | + | + | + |  | + |  | + |  |
| *Anax imperator* |  |  |  |  |  |  |  |  |  |  |  |  |  |  |  |  |  |  |  |  |  |  |  |  |  |  |  |  | + |  |
| Anisoptera (I + II instar) | + | + |  |  |  | + |  | + | + | + |  | + |  | + | + |  | + |  |  | + | + |  | + |  | + |  | + |  |  |  |
| *Coenagrion puella* | + |  |  |  |  |  |  | + |  | + |  | + |  | + |  |  |  | + |  | + |  |  |  |  | + |  |  |  |  |  |
| *Enallagma cyathigerum* | + |  |  |  |  |  |  |  |  |  |  | + |  |  |  |  |  |  |  |  | + |  | + |  |  |  |  |  |  |  |
| *Erythromma najas* | + | + |  |  |  |  |  |  |  |  |  | + |  | + |  |  |  | + |  |  |  | + |  |  |  |  | + |  |  |  |
| *Ischnura elegans* | + | + |  |  |  |  |  | + |  | + |  | + |  | + |  |  | + | + |  | + | + | + | + | + | + | + | + |  | + |  |
| *Lestes sponsa* |  |  |  |  |  |  |  |  |  |  |  |  |  |  |  |  |  |  |  |  |  |  |  |  |  |  |  |  | + |  |
| *Libellula depressa* |  |  |  |  |  |  |  |  |  | + |  |  |  |  |  |  |  |  |  |  |  |  |  |  |  |  |  |  |  |  |
| *Libellula quadrimaculata* |  |  |  |  |  |  |  |  |  |  |  |  |  |  |  |  |  |  |  |  | + |  |  |  |  |  |  |  |  |  |
| *Orthetrum cancellatum* |  |  |  |  |  |  |  |  |  |  |  |  |  |  |  |  |  |  |  |  |  |  |  |  | + |  |  |  |  |  |
| *Pyrrhosoma nymphula* |  |  |  |  |  |  |  |  |  |  |  |  |  |  |  |  |  |  |  | + |  |  |  |  | + |  |  |  |  |  |
| *Sympetrum fonscolombii* |  |  |  |  |  |  |  |  |  | + |  |  |  |  |  |  |  |  |  |  |  |  |  |  |  |  |  |  |  |  |
| *Sympetrum sanguineum* | + | + |  |  |  |  |  |  |  | + |  | + |  | + |  |  |  |  |  |  | + |  | + |  |  |  |  |  |  |  |
| *Sympetrum striolatum* | + |  |  |  |  |  |  |  |  | + |  |  |  | + |  |  |  |  |  |  |  |  |  |  |  |  |  |  |  |  |
| Zygoptera (I +II instar) | + | + |  |  |  | + |  | + |  | + |  | + |  | + |  |  | + | + |  | + | + | + | + | + | + |  | + |  | + |  |
| **Trichoptera** |  |  |  |  |  |  |  |  |  |  |  |  |  |  |  |  |  |  |  |  |  |  |  |  |  |  |  |  |  |  |
| *Agraylea multipunctata* | + | + |  |  |  |  |  |  | + | + |  | + |  |  |  |  |  | + | + |  | + |  |  |  | + |  | + |  | + |  |
| *Agraylea sexmaculata* |  | + |  |  |  |  |  |  |  | + |  | + |  |  |  |  |  |  |  |  | + |  |  |  |  |  |  |  |  |  |
| *Agrypnia pagetana* | + |  |  |  |  |  |  |  |  |  |  | + |  |  |  |  | + | + |  |  |  |  |  |  |  |  |  |  |  |  |
| *Anabolia nervosa* |  |  |  |  |  |  |  |  |  |  |  |  |  |  |  |  |  | + |  |  |  |  |  |  | + |  |  |  |  |  |
| *Athripsodes aterrimus* |  |  |  |  |  |  |  |  |  |  |  |  |  |  |  |  |  | + |  |  |  |  | + |  |  |  |  |  |  |  |
| *Cyrnus flavidus* | + |  |  |  |  |  |  |  |  |  |  |  |  |  |  |  |  | + |  |  |  |  | + |  | + |  |  |  |  |  |
| *Cyrnus trimaculatus* |  |  |  |  |  |  |  |  |  | + |  |  |  |  |  |  |  |  |  |  |  |  |  |  |  |  |  |  |  |  |
| *Holocentropus dubius* | + |  |  |  |  |  |  |  |  |  |  |  |  |  |  |  |  | + |  |  |  |  |  |  |  |  |  |  |  |  |
| *Holocentropus picicornis* | + |  |  |  |  |  |  |  |  |  |  | + |  | + |  |  |  | + |  |  |  |  |  |  | + |  |  |  |  |  |
| *Hydroptila* spp. | + | + |  |  |  |  |  |  | + | + |  |  |  | + |  |  |  |  |  |  |  |  | + |  | + |  |  |  |  |  |
| *Leptocerus tineiformis* | + | + |  |  |  |  |  |  |  |  |  | + |  |  |  |  |  | + |  |  |  |  |  |  |  |  | + |  |  |  |
| Limnephilidae spp. |  |  |  |  |  |  |  |  |  |  |  |  |  |  |  |  |  |  |  |  |  |  | + |  |  |  |  |  |  |  |
| *Limnephilus decipiens* |  | + |  |  |  |  |  |  |  |  |  |  |  |  |  |  |  | + |  |  |  |  |  |  |  |  |  |  |  |  |
| *Limnephilus flavicornis* |  |  |  |  |  |  |  | + |  |  |  |  |  | + |  |  |  | + |  |  |  |  |  |  |  |  | + | + |  |  |
| *Limnephilus lunatus* |  | + |  |  |  |  |  | + | + | + |  | + |  | + |  |  |  | + |  | + | + |  | + | + | + |  | + | + | + | + |
| *Limnephilus marmoratus* |  |  |  |  |  |  |  |  |  |  |  |  |  | + |  |  |  |  |  |  |  |  |  |  |  |  | + |  |  |  |
| *Lype reducta* |  | + |  |  |  |  |  |  |  |  |  |  |  |  |  |  |  |  |  |  |  |  |  |  |  |  |  |  |  |  |
| *Molanna angustata* |  |  |  |  |  |  |  |  |  |  |  |  |  |  |  |  |  | + |  |  |  |  | + | + |  |  |  |  |  |  |
| *Mystacides azurea* |  |  |  |  |  |  |  |  |  |  |  |  |  |  |  |  |  |  |  |  |  |  |  |  | + |  |  |  |  |  |
| *Mystacides longicornis/nigra* | + | + |  |  |  | + |  |  | + |  |  | + |  |  |  |  | + |  |  |  | + |  | + | + | + |  |  |  |  |  |
| *Oecetis lacustris* |  |  |  |  |  |  |  |  |  |  |  |  |  |  |  |  |  |  |  |  |  |  |  |  | + |  |  |  |  |  |
| *Oecetis ochracea* |  |  |  |  |  |  |  |  |  |  |  |  |  |  |  |  |  |  |  |  | + |  |  |  |  |  |  |  |  |  |
| *Oxyethira* spp. | + | + |  |  |  |  |  |  |  |  |  |  |  |  |  |  |  | + |  |  |  |  |  |  | + |  | + |  |  |  |
| *Phryganea bipunctata* |  | + |  |  |  |  |  |  |  | + |  | + |  | + |  |  |  | + |  |  |  |  | + |  | + |  | + |  |  |  |
| *Triaenodes bicolor* |  | + |  |  |  |  |  |  |  |  |  | + |  | + |  |  |  | + |  |  | + | + | + |  | + |  |  |  | + |  |
| **Tricladida** |  |  |  |  |  |  |  |  |  |  |  |  |  |  |  |  |  |  |  |  |  |  |  |  |  |  |  |  |  |  |
| *Dendrocoelum lacteum* |  |  |  |  |  |  |  | + | + | + |  |  | + | + |  |  |  | + |  |  |  | + | + |  |  |  | + | + | + |  |
| *Dugesia lugubris/polychroa* | + |  |  |  |  | + | + | + |  |  |  |  |  | + |  | + | + | + | + | + | + | + | + | + |  | + |  |  | + |  |
| *Dugesia* spp. | + |  |  |  |  |  |  |  |  |  |  |  |  |  |  |  |  |  |  |  |  |  |  |  |  |  |  |  |  |  |
| *Dugesia tigrina* | + | + |  |  |  | + |  | + | + | + |  | + |  | + |  |  | + | + | + | + | + | + |  |  | + | + |  |  |  |  |
| *Planaria torva* |  |  |  |  |  |  |  |  |  |  |  |  |  |  |  |  |  |  |  |  |  |  |  |  |  | + |  |  |  |  |
| *Polycelis nigra/tenuis* |  |  |  |  | + |  |  | + | + |  |  |  |  |  |  |  |  |  |  | + | + | + | + |  |  |  |  |  | + |  |
| Tricladida spp. | + |  |  |  | + |  |  |  |  |  |  |  |  |  |  |  | + | + | + |  |  | + | + |  | + | + | + |  | + | + |

T3. Rare and notable macroinvertebrate species found during the study and number of sites at which the species was found within each pond type

| Species | Order | Family | RDB^1^ | NB^2^ | NR^3^ | CCI^4^ | Type1 | Type 2 | Type 3 | Type 4 |
| --- | --- | --- | --- | --- | --- | --- | --- | --- | --- | --- |
| *Hydrochus elongatus* | Coleoptera | Hydrochidae | ● |  |  | 8 | 1 |  |  |  |
| *Helochares lividus* | Coleoptera | Hydrophilidae |  | ● |  | 7 | 2 |  |  |  |
| *Rhantus suturalis* | Coleoptera | Dytiscidae |  | ● |  | 7 |  |  | 1 |  |
| *Cercyon convexiusculus* | Coleoptera | Sphaeridiidae |  | ● |  | 7 | 1 |  |  |  |
| *Hydroglyphus geminus* | Coleoptera | Dytiscidae |  | ● |  | 7 |  | 1 |  | 1 |
| *Mesovelia furcata* | Hemiptera | Mesoveliidae |  |  | ● | 6 | 1 |  |  |  |
| *Limnephilus decipiens* | Trichoptera | Limnephilidae |  |  | ● | 6 | 2 |  |  |  |
| *Micronecta scholtzi* | Hemiptera | Corixidae |  |  | ● | 6 | 5 | 2 | 1 |  |

^1^Red Data Book category 3 – nationally rare

^2^Notable B; thought to occur in 31-100 10km grid squares within the U.K.

^3^Notable Regional; found in five or fewer U.K. localities

^4^Community Conservation Score (after Chadd & Extence 2004)
